# HIV-1 and Its gp120 Inhibits the Influenza A(H1N1)pdm09 Life Cycle in an IFITM3-Dependent Fashion

**DOI:** 10.1371/journal.pone.0101056

**Published:** 2014-06-30

**Authors:** Milene Mesquita, Natalia Fintelman-Rodrigues, Carolina Q. Sacramento, Juliana L. Abrantes, Eduardo Costa, Jairo R. Temerozo, Marilda M. Siqueira, Dumith Chequer Bou-Habib, Thiago Moreno L. Souza

**Affiliations:** 1 Respiratory Viruses Laboratory, WHO/NIC, Oswaldo Cruz Institute/Fiocruz, Rio de Janeiro, RJ, Brazil; 2 Laboratory on Thymus Research, Oswaldo Cruz Institute/Fiocruz, Rio de Janeiro, RJ, Brazil; University of Georgia, United States of America

## Abstract

HIV-1-infected patients co-infected with A(H1N1)pdm09 surprisingly presented benign clinical outcome. The knowledge that HIV-1 changes the host homeostatic equilibrium, which may favor the patient resistance to some co-pathogens, prompted us to investigate whether HIV-1 infection could influence A(H1N1)pdm09 life cycle *in vitro*. We show here that exposure of A(H1N1)pdm09-infected epithelial cells to HIV-1 viral particles or its gp120 enhanced by 25% the IFITM3 content, resulting in a decrease in influenza replication. This event was dependent on toll-like receptor 2 and 4. Moreover, knockdown of IFITM3 prevented HIV-1 ability to inhibit A(H1N1)pdm09 replication. HIV-1 infection also increased IFITM3 levels in human primary macrophages by almost 100%. Consequently, the arrival of influenza ribonucleoproteins (RNPs) to nucleus of macrophages was inhibited, as evaluated by different approaches. Reduction of influenza RNPs entry into the nucleus tolled A(H1N1)pdm09 life cycle in macrophages earlier than usual, limiting influenza's ability to induce TNF-α. As judged by analysis of the influenza hemagglutin (HA) gene from *in vitro* experiments and from samples of HIV-1/A(H1N1)pdm09 co-infected individuals, the HIV-1-induced reduction of influenza replication resulted in delayed viral evolution. Our results may provide insights on the mechanisms that may have attenuated the clinical course of Influenza in HIV-1/A(H1N1)pdm09 co-infected patients during the recent influenza form 2009/2010.

## Introduction

Acute respiratory tract infections have great impact on public health, since they are a major cause of morbidity and mortality [1]. Among the respiratory viruses, influenza is one of the most important pathogens due to their seasonality and continued pandemic threat [2]. This negative-sense-RNA orthomixovirus enters the cells by binding to sialic acid residues, followed by endocytosis and fusion of viral envelope with endocytic membrane. Viral ribonucleoproteins (RNP), composed of RNA polymerase complex, viral RNA, nucleoprotein (NP) and nuclear export proteins (NEP), are released and transported to the cell nucleus where transcription and replication of the viral genome takes place. Next, viral RNPs are transported from the nucleus to the host cell plasma membrane, where the final steps of virus assembly and virion budding occur. The active influenza replication cycle is carried out in the human epithelial surface of the respiratory tract, which represents the largest interface for gas exchange, and plays a key role in fluid and ion transport [3] as well as surfactant production [4].

Influenza infection also triggers the homing of blood monocytes to the respiratory tract [5], where they differentiate into macrophages capable of producing inflammatory mediators, such as the cytokines, type I Interferons (IFN-1) and tumor necrosis factor-α (TNF-α), and the chemokine CCL5 [6], [7] in response to infection [8]. Macrophages are susceptible to influenza infection and viral proteins are made, but no infectious virus progeny is made [9]. Thus, influenza-infected macrophages are endowed with a janus-face: they orchestrate the host immune response [10], but may release cytokine in an atypical fashion – leading to influenza-induced cytokine storm [11]. Polarization to one of these conditions could be highly influenced by the presence of different stimuli in the respiratory tract, such as pathogen-associated molecular patterns (PAMPs) which could activate toll-like receptors (TLR) [8], [12], [13].

The infection by the human immunodeficiency virus type 1 (HIV-1), the causal agent of the acquired immunodeficiency syndrome (AIDS) affects approximately 34 million people worldwide, and this number continues to grow [14]. HIV-1 infection provokes a progressive loss of CD4^+^ T cells through virus-, cellular- and immune activation-dependent mechanisms [15], eventually collapsing the immune system. HIV-1 imposes a new homeostatic equilibrium for its human host [15], [16], as it replicates in different anatomical compartments, such as the gastrointestinal tract, lymph nodes and lungs, establishing numerous virus sanctuaries [17], [18]. HIV-1 surface glycoprotein gp120 and other viral products may even affect cells not susceptible to HIV-1 infection, as they have promiscuous binding to numerous cell-surface molecules – triggering different events, such as cell death [19]. Whereas the HIV-1-host cell interactions and tissue abnormalities at the gut-associated lymphoid tissue (GALT) are well known [17], [20], the fate of HIV-1 infection and replication in the respiratory tract remains poorly understood [21], [22].

In contrast to the increased mortality observed in immunocompromised individuals [23], such as cancer and transplanted patients [24], affected by the emergence of influenza A(H1N1)pdm09, HIV-1-infected individuals presented benign clinical outcomes [25]–[30]. For instance, Barchi *et al* found that the clinical outcome in HIV-1-infected individuals with A(H1N1)pdm09 infection was not different from that in immunocompetent patients [25], and Ormsby *et al* reported that only HIV-1-infected subjects that had progressed to the immune deficiency typical of AIDS presented an increased mortality by A(H1N1)pdm09 [29]. Therefore, it is plausible to hypothesize that during HIV-1/influenza co-infection a new homeostatic balance must be imposed on the host. For example, components of the innate immunity have proved to significantly contribute to the effective control of viral infections, such as interferon-induced cellular restriction factors (e.g. Interferon-induced transmembrane protein 3; IFITM3) that may target both HIV-1 and influenza A [31]–[35]. In this study, we show that influenza replication is attenuated during HIV-1/A(H1N1)pdm09 co-infection experimental assays, as HIV-1 viral particle or its viral surface glycoprotein gp120 reduce A(H1N1)pdm09 replication in an IFITM3-dependent fashion. Consistent with HIV-1-induced decreased influenza replication, A(H1N1)pdm09 evolution was delayed in the presence of HIV-1, both in *in vitro* experiments and in individuals co-infected with both viruses, a phenomenon that may correlate with mild influenza outcomes during clinical episodes of co-infection. Thus, we report here, for the first time, that HIV-1 interferes with the A(H1N1)pdm09 replicative cycle, arresting the influenza evolution and attenuating the viral replication. Our results may contribute to explain the mild influenza infection observed in patients co-infected by HIV-1 and influenza A(H1N1)pdm09.

## Materials and Methods

### Ethics statement

The Research Ethics Committee of the Evandro Chagas Clinical Research Institute (http://www.ipec.fiocruz.br/cgi/cgilua.exe/sys/start.htm?sid=17, led by Dr. Léa Camillo-Coura) approved the retrospective use of samples and clinical data from HIV-1/A(H1N1)pdm09 co-infected individuals under protocol number 00930112.7.0000.5262. Need for informed consent has been waived. All data were analysed in an anonymous fashion.

### Cells and Viruses

Human epithelial cervical cancer (HeLa) and Madin-Darby Canine Kidney (MDCK) cells were cultured with Dulbecco's Modified Eagle's Medium (DMEM; LGC Biotecnologia, São Paulo, Brazil) and Minimum Essential Eagle's Medium (MEM; LGC Biotecnologia), respectively, supplemented with 10% fetal bovine serum (FBS; HyClone, Logan, UT, USA), 100 U/mL penicillin and 100 mg/mL streptomycin (Sigma-Aldrich). Cells were cultured at 37°C in 5% CO_2_ atmosphere. Monocyte–derived macrophages were obtained through plastic adherence of peripheral blood mononuclear cells (PBMCs) previously isolated by density gradient centrifugation (Ficoll-Paque, GE Healthcare) from buffy coat preparations of blood from healthy donors. Briefly, 2.0×10^6^ PBMCs were plated onto 48-well plates (NalgeNunc) in DMEM containing 10% human serum (HS; Milliipore) and penicillin/streptomycin. Cells were maintained at standard culture conditions for 6–7 days for monocyte differentiation into macrophages. Then, non-adherent cells were washed out, and the remaining macrophage layer was maintained in DMEM with 5% HS. Macrophage purity was above 95%, as determined by flow cytometric analysis (FACScan; Becton Dickinson) using anti-CD3 (BD Biosciences) and anti-CD16 (Southern Biotech) monoclonal antibodies. For some assays, macrophages were prepared in 25-cm^2^ plastic culture bottles, from 4.0×10^7^ PBMCs previously seeded in 5 mL of medium per bottle.

Cell infection assays were performed with influenza A(H1N1)pdm09 (A/Minas Gerais/490/2009 strain, which has been isolated from a Brazilian patient at the Brazilian National Influenza Center/WHO during the 2009 pandemics; this isolate is an A/California/07/2009-like strain) or with the monocytotropic, CCR5-dependent isolate HIV-1_Ba-L_ (donated by the AIDS Research and Reference Reagent Program, Division of AIDS, National Institute of Allergy and Infectious Diseases, National Institutes of Health; NIH, Bethesda, MD). The Influenza virus was expanded in MDCK cells [36], and the HIV-1 isolate was expanded in phytohemagglutinin-activated PBMCs [37]. The viral stocks were aliquoted and stored at −70°C for further studies.

### Cell Viability

The effect of A(H1N1)pdm09 virus infection on macrophage or HeLa cells viability was evaluated after 72 h of cell exposure to different virus MOIs (1, 5 and 10) using the reduction of 2,3-Bis (2-methoxy-4-nitro-5-sulfophenyl)-2*H*-tetrazolium-5-carboxanilide inner salt (XTT; Sigma) assay, as described[38]. Conditioned medium for influenza growth (mock) was used as a control, in same proportions as the influenza-containing medium. Additionally, the viability of HeLa cells exposed to the same conditions was also determined by Trypan blue dye exclusion assay, at 0.04%.The viability of HeLa cells and macrophages was ≥95% under the conditions described above.

### HIV-1 and A(H1N1)pdm09 co-infections

Macrophages were infected with HIV-1 by exposing them overnight to viral suspensions containing 10 ng/mL of p24 antigen (p24 Ag). Non-internalized viruses were then removed by washing, and cell monolayers were replenished with fresh medium. Every 7 days or when necessary, approximately 50% of the medium was replaced by fresh medium. Influenza A(H1N1)pdm09 or the corresponding mock infection were added to uninfected or to HIV-1-infected macrophages 10 days after HIV-1 infection, at different MOIs and periods of time. Alternatively, HeLa cells seeded in 24-well plates (5.0×10^5^ cells/well) were infected with A(H1N1)pdm09 at different MOIs for 1 h at 37°C and 5% CO_2_. Then, the monolayers were treated with DMEM containing 10% FBS and HIV-1 suspensions (10 ng/mL of p24 Ag; unless otherwise mentioned), HIV-1 recombinant gp120 (5 µg/mL), Tat (100 ng/mL), oxidized Tat (Tat-ox) [39] or IFN-γ (10 ng/mL). The Influenza titers were quantified in the cell supernatant 3 days after infection. For some specific experiments, influenza-infected or -uninfected HeLa cells were treated with anti-TLR2 (aTLR2), anti-TLR4 (aTLR4), control isotype, soluble CD4 (sCD4) or the antiretroviral enfuvirtide (T20) for 15 to 30 minutes prior to exposure to HIV-1. Antibodies were purchased from Invivogen and used at 0.5 µg/mL. Blockers of HIV-1 entry have been received by donation from NIH AIDS Reagent program. T20 and sCD4 were used at 30 nM and 1 µg/mL, respectively.

### HIV-1 and Influenza quantifications

HIV-1 production was evaluated in cell-culture supernatants by a commercial enzyme-linked immunosorbent assay (ELISA) kit according to the manufacturer's instructions (ZeptoMetrix). HIV-1 RNA was detected and as described elsewhere [40].

The titers of Influenza in the supernatant of the cultures was measured in MDCK cells [36] and quantified by real time RT-PCR. All reagents for real time RT-PCR, including primers, probes and enzymes, were used as recommended by WHO [36]. Virus quantification was based on a standard curve method, as described elsewhere with some modifications [41]. In brief, real time RT-PCR with RNA from experimental assays was run in parallel with serial 10-fold dilutions of PET26b+ plasmid (Novagen) containing influenza HA or M1 synthetic gene inserts (Genescript) flanked between XhoI and HindII restriction sites. Then, quantification was expressed as copies/mL (http://cels.uri.edu/gsc/cndna.html). Negative controls without template were included in all reactions.

To quantify total influenza RNA and mRNA, A(H1N1)pdm09-infected macrophages were lysed 24 h p.i. and RNA extracted with a commercial kit (RNeasy mini kit; Qiagen). CDNA was synthetized with SuperScript III (Life Technologies) using oligo.dT (Life Technologies) or UNI-12 (5′-AGCRAAAGCAGG-3′) as the primers for first-strand synthesis, for 1 h at 45°C. Oligo.dT- and UNI-12 primers allow preferentially the retrotranscription mRNA and total influenza RNA, respectively. Real time PCR assays were performed to amplify influenza genes M1 or HA genes [41], [42]. For a control, total RNA and mRNA were measured in influenza-infected macrophages treated with ribavirin (kidney donated from Dr. Nubia Boechat, Farmamginhos, Fiocruz).

### Western Blotting

Immunoblotting assays were performed to quantify IFITM3 contents in HeLa cells and macrophages exposed to HIV-1 (10 ng/mL of p24 Ag), rHIV-1gp120 (5 µg/mL) or IFN-γ (10 ng/mL) and kept at standard culture conditions during different periods of time. Cells were then lysed with Laemmli's buffer (1 mM Tris-HCl, pH 6.8; 0.02% bromophenol blue; 50 mM *b*-mercaptoethanol; 10% SDS; 10% glycerol), and a 20 µg aliquot of the extracted proteins (Qubit Protein assay kit, Invitrogen) was separated by 12% sodium dodecyl sulfate–polyacrylamide gel electrophoresis and transferred to polyvinylidene fluoride filters (PVDF, Hybond-C). Filters were then blocked and incubated with rabbit polyclonal anti-IFITM3 (Santa Cruz Biotechnology) or mouse polyclonal anti-β-actin antibody (Sigma) for 1 h. Specific reactive proteins were detected with the enhanced chemiluminescence method using an ECL kit (GE Heatlhcare). We also performed densitometry analysis of the blots using ImageJ software (Version 1.6.0). Densitometry values were initially expressed as arbitrary units to calculate mean ± SEM. Next, densitometry values for the control bands (medium only) were normalized to be 1.0. Therefore, the values for the other bands were expressed as n-fold change, throughout of this article.

### Knockdown of IFITM3

To evaluate the biological role of IFITM3 during HIV-1 and A(H1N1)pdm09 co-infection, we performed siRNA assays as previously described [43]. Briefly, 6.0×10^4^ HeLa cells plated onto 24-well plates. Cells were transfected with siRNA for IFITM3 (Life Technologies) or its scramble sequence at a concentration of 0.4 nM in OPTI-MEM, using lipofectamine 2000 (Sigma). After 6 h of transfection, the cells were washed and infected with A(H1N1)pdm09 (MOI  = 1) for 1h. Then, cells were exposed to HIV-1 overnight. The culture supernatants were harvested and A(H1N1)pdm09 quantified by qRT-PCR. In parallel, cell monolayers were lysed with Laemmli's buffer, and western blotting to IFITM3 and β-actin was performed.

### Measurements of A(H1N1)pdm09 binding and entry

HIV-1-infected macrophages (10 days of infection) were inoculated with A(H1N1)pdm09 (MOI  = 1) during 1 h at 4°C, a condition that allows only virus adsorption. Then, cultures were washed with PBS and lysed with buffer A (10 mM HEPES, 1.5 mM MgCl_2_, 10 mMKCl, 0.5% NP-40, 1 mM DTT, 0.5 mM PMSF). In parallel assays, after the 4°C-incubation, cells were washed and incubated with warm medium during 6 h at 37°C, followed by lysing with buffer A. This last condition is enough to allow influenza RNPs to reach the cell nucleus [44]. We defined these samples as early lysates because they were obtained before the completion of one replicative cycle.

Another set of cells were instead lysed at later time points. That is, cells were initially infected as described above, at 4°C, washed and incubated with warm culture medium for 24 h at 37°C. After 24 h p.i., cells were lysed with buffer A. Both early and late lysates were centrifuged (10 min. at 1000× *g*), allowing the separation of nuclear (pellet) and non-nuclear fractions (supernatant).

Considering that an end-point analysis of our study is arrival of influenza RNPs into the nuclei of macrophages, we confirmed the purity of our nuclear RNA preparation. To do so, nuclear fraction was prepared as described above and the expression of the housekeeping genes GAPDH, RNAse P, β-actin and long non-coding RNA (lncRNA) analyzed. In brief, RNA extraction was performed with RNeasy mini kit. CDNA was synthetized with SuperScript III using oligo.dT as the primer, as described above. To perform the real time PCR assay, syber green master mix was used, with 1.5 µL of cDNA and cycling conditions described elsewhere [45]. Primers are described in Table S1. The lncRNA is exclusively expressed in the nuclear fraction, based on the literature [46] and our results (Figure S1). This means that cross-contamination between RNA from non-nuclear fraction on the nuclear preparation is unlike to occur.

### Cell preparations and co-infection for immunofluorescence assays

PBMCs (2×10^6^ cells) were seeded onto Aclar plastic coverslipes (Pro-Plastics Inc., Linden, NJ, USA) for 3 h on 24-well plates previously coated with rat-tail collagen. After this period of time, unadherent cells were washed out. Macrophages were allowed to differentiate for 7 days, when they were infected with HIV-1. At the 12^nd^ day after HIV-1 infection, cells were infected with influenza at MOI of 5 for different periods of time.

### Immunofluorescence microscopy and digital image acquisition

Cells were washed with PBS and fixed with 4% paraformaldehyde for 10 min. After that, they were permeabilized with 0.5% Triton-X 100 for 10 min. Primary antibodies (anti-HIV-1 p24, mouse IgG, NIH AIDS Reagents program; and anti-influenza NP, rabbit IgG, AbCam, USA) were incubated for 1 h at 37°C. After incubation, cells were rinsed three times and incubated with Alexa Fluor 488- or Alexa Fluor 546-conjugated secondary antibodies (goat anti-rabbit and goat anti-mouse IgGs; Molecular Probes, USA) for 1 h at 37°C. Nuclei were stained with DAPI (0.1 µg/ml in 0.9% NaCl). After washing for 5 min with 0.9% NaCl, specimens were mounted in glycerol with 5% n-propyl gallate, 0.25% DABCO (1,4–diazabicyclo(2,2,2)octane) and 0.0025% para-phenylenediamine (all from Sigma). Images were analyzed with Fiji software (ImageJ, http://imageJ.nih.gov/ij/). As a control, experiments were performed without antibodies to monitor any background signs. Aclar coverslips were examined in an Axio Imager A2 microscope with phase-contrast optics (Carl Zeiss, Germany), and images were acquired with an Olympus DP72 digital camera (Olympus, Japan).

### Patients and data collection

Nasopharingeal aspirates or swabs (NPAs) from patients with confirmed diagnosis of A(H1N1)pdm09, co-infected or not by HIV-1, were collected from individuals attending the Brazilian National influenza surveillance program in different regions of the country. The collected NPAs were sent to the Brazilian National Influenza Center were the laboratory-based diagnosis were confirmed by real time RT-PCR assay [36].

### A(H1N1)pdm09 Hemagglutinin (HA) gene sequencing and phylogenetic analysis

RNA was extracted from cell culture supernatants or from NPAs and subjected to one-step RT-PCR using Superscript III/Platinum Taq, as previously described [36]. The RT-PCR products were purified and the sequencing was performed by the Sanger method, using the “Big Dye Terminator Cycle Sequencing Ready Reaction” kit (Applied Biosystems). After that, fragments were analyzed using an automatic sequencer (ABI PRISMTM 3130XL-avant Genetic Analyzer; PE Applied Biosystems). Consensus sequences for HA genes were generated in Seqscape software (Applied Biosystems) and aligned to other sequences deposited in GenBank using the ClustalW algorithm in Megalign (Mega software 5.2). Sequences were analyzed using neighbor-joining with bootstrap (1,000 times) using the Mega 5.2 suite of programs. Bootstrap values greater than 70 were considered significant.

### Statistical analysis

The results of this study were prepared using Excel 2010 for Windows software (Microsoft), from at least three independent experiments. Means ± standard error of the means (SEM) are displayed. Statistical analysis calculations were performed using the same software, and the comparisons between means were considered significantly different when the *P* value was less than 0.05, by means of Student's*t* test.

## Results

### HIV-1 and its surface glycoprotein gp120 trigger an Interferon-like inhibition of A(H1N1)pdm09 replication in epithelial cells

**Figure 1:**
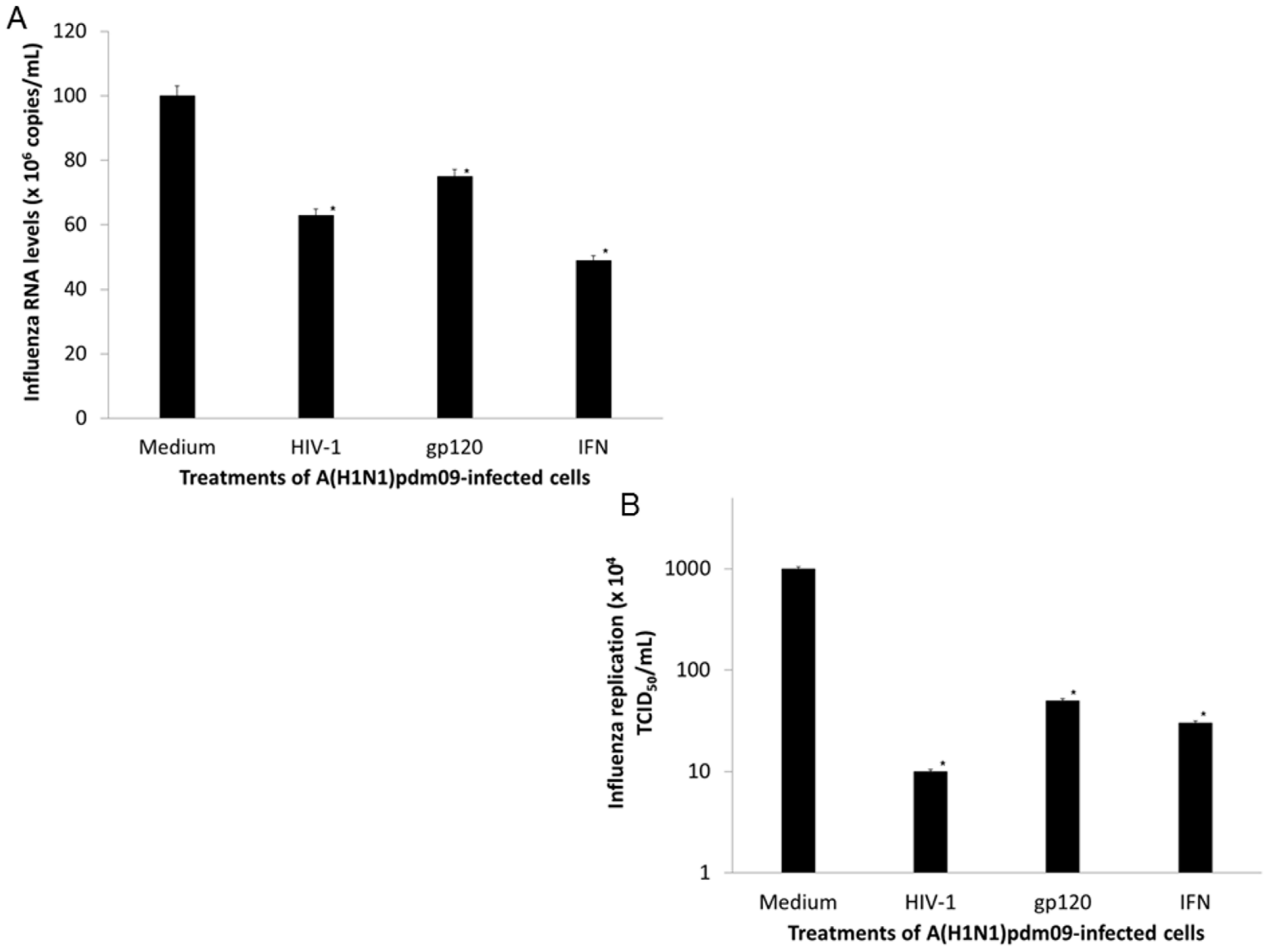
The exposure to HIV-1 or to HIV-1 gp120 reduces influenza A(H1N1)pdm09 replication. A(H1N1)pdm09-infected HeLa cells were exposed to HIV-1 (10 ng/mL of HIV-1 p24 Ag), gp120 (5 µg/mL) or IFN-γ (10 ng/mL). After 24 h, the RNA was extracted from the culture supernatants, and the influenza virus was quantified by qRT-PCR (**A**) or supernatants were tittered in MDCKs (**B**). The IFN-γ was used as positive control. The asterisks indicate statistical significance (*P*<0.05) over control (medium). (n = 4).

Influenza replicates in epithelial cells of the respiratory tract [47]. Influenza-infected tissues release β-chemokines, such as CCL5 (Rantes), which is chemotactic for blood monocytes [7]. Monocytes migrate to the respiratory tract where they differentiate into macrophages and may participate during the host immune response to influenza infection [7]. In HIV-1-infected individuals, macrophages sustain viral replication even in the presence of antiretroviral therapy [21], [22]. Because HIV-1 and influenza infect macrophages and epithelial cells in the respiratory tract [15], [47], respectively, it is thus plausible that influenza-infected cells are exposed to HIV-1-derived products and/or viral particles at the same anatomical site. To evaluate whether HIV-1 could change influenza replication, we exposed A(H1N1)pdm09-infected HeLa cells to HIV-1 or to its surface glycoprotein gp120. An approximately 40% and 25% decrease in the quantification of A(H1N1)pdm09 was observed when influenza-infected cells were exposed to HIV-1 or to gp120, respectively (Figure 1A). Infectivity of influenza virus produced in the presence of HIV-1 and its gp120 was also reduced by approximately 2-log_10_ (Figure 1B). Of note, other HIV-1 proteins, which could be secreted from HIV-1-infected cells, such as Tat, did not affect influenza replication significantly (Figure S2A and B). Moreover, the HIV-1 effects towards influenza replication were dependent on the retroviral input (Figure S3). Inhibition of influenza replication was not due to exposure of HeLa cells to different agents, because cells exposed HIV-1 or gp120 remained with over 95% of viability (Figure S4). Our results therefore suggest that HIV-1 or its products presented in extracellular milieu may weaken influenza replication. As expected, there was no HIV-1 replication in HeLa cells (Figure S5). Interferon-γ (IFN-γ), a known inhibitor of influenza replication [48], was used as a positive control to inhibit A(H1N1)pdm09 replication (Figure 1).

### HIV-1-triggered inhibition of influenza replication is TLR2/4-dependent and requires the engagement of the restriction factor IFITM3

**Figure 2:**
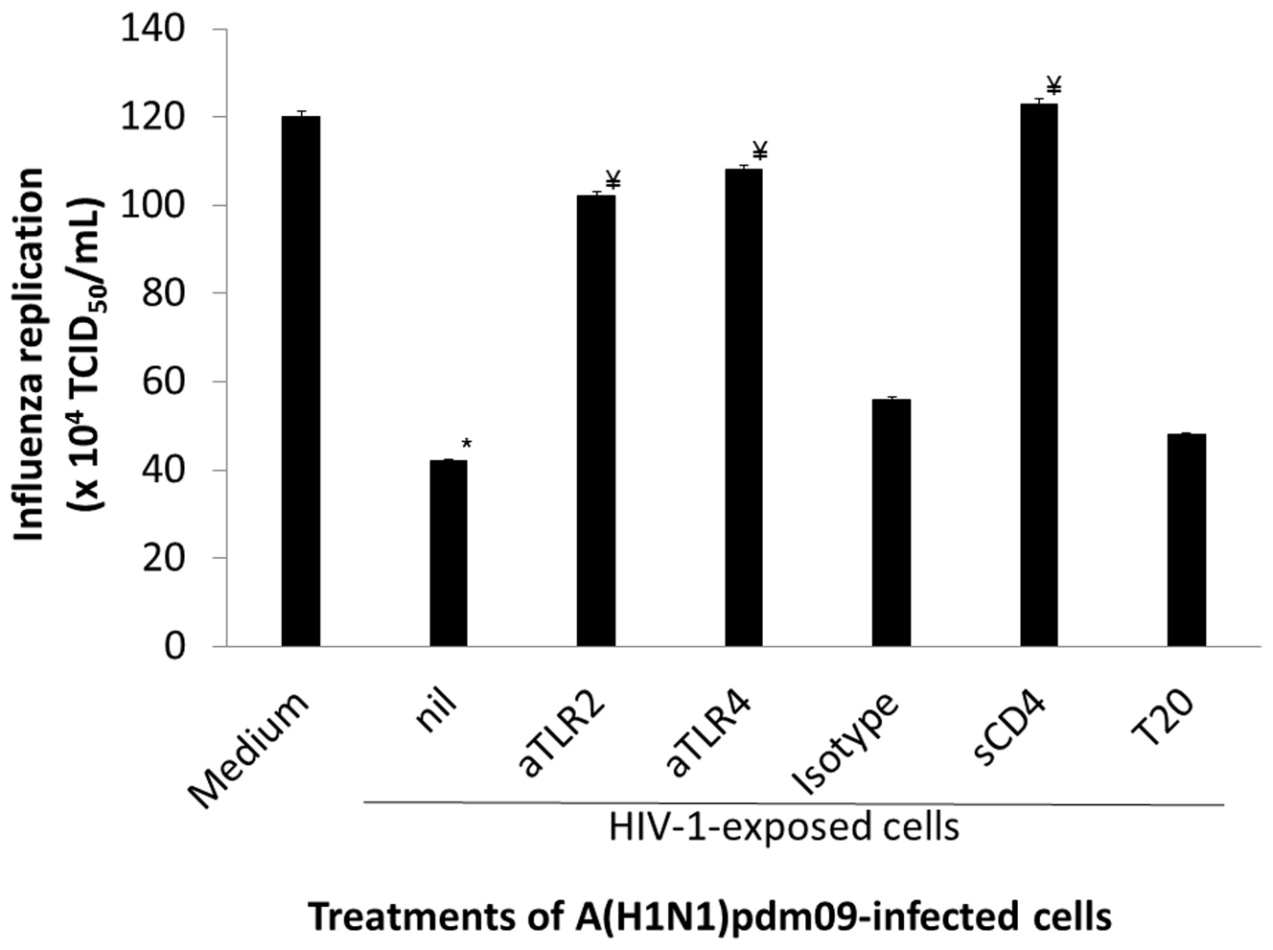
Inhibition of influenza A(H1N1)pdm09 replication by HIV-1 is prevented by anti-TLR 2 and 4 antibodies and sCD4. A(H1N1)pdm09-infected HeLa cells were incubated with blocking antibodies anti-TLR2 (aTLR2), -TLR4 (aTLR4), isotype control, sCD4 or T20 15 to 30 min, prior to exposure to HIV-1. After 24 h, the A(H1N1)pdm09 virus in supernatants were tittered in MDCKs. * *P*<0.05 over control (medium). ^#^ *P*<0.05 over HIV-1-exposed cells (nil). (n = 4)

Although HIV-1 entry is dependent upon interaction between viral gp120 with CD4 and CCR5 or CXCR4 co-receptors, this viral glycoprotein may bind to multiple molecules on the cell surface. Recently, it has been demonstrated that in epithelial cells HIV-1 gp120 may bind to TLR 2 and 4 [13]. The agonism of TLR 2 and 4 in the respiratory tract may lead to an IFN-I-dependent response [12]. It has been demonstrated that IFNs inhibit influenza replication in cell culture and in laboratory animals by the restriction factor IFITM3 [49]. Since we showed above that Influenza production is diminished upon exposure of A(H1N1)pdm09-infected cells to IFN-γ, HIV-1 or HIV-1 gp120 (Figure 1), we next evaluated whether the HIV-1-induced inhibition of influenza replication would be dependent on TLR 2 and/or 4. Influenza-infected HeLa cells were exposed to HIV-1 in the presence of blocking antibodies to TLR 2 or 4. As we can see in Figure 2, both antibodies prevented HIV-1's ability to inhibit influenza replication. Of note, control isotype antibody had no influence on HIV-1-dependent impact over influenza replication.

Importantly, sCD4 prevented the HIV-1-dependent inhibition of influenza replication, whereas T20 did not (Figure 2). Although both treatments prevent HIV-1 entry, only sCD4 could block gp120 interaction with virtually any receptor on the cell surface. On the other hand, T20 blocks HIV-1 envelope fusion with the plasma membrane by preventing gp41 translocation – which means that viral gp120 is free to interact with TLR 2 and 4 under treatment with T20. These results reinforce that the surface glycoprotein from HIV-1 virions may indeed interact with non-classical receptors to influence the physiology of cells not susceptible to HIV-1 infection.

**Figure 3:**
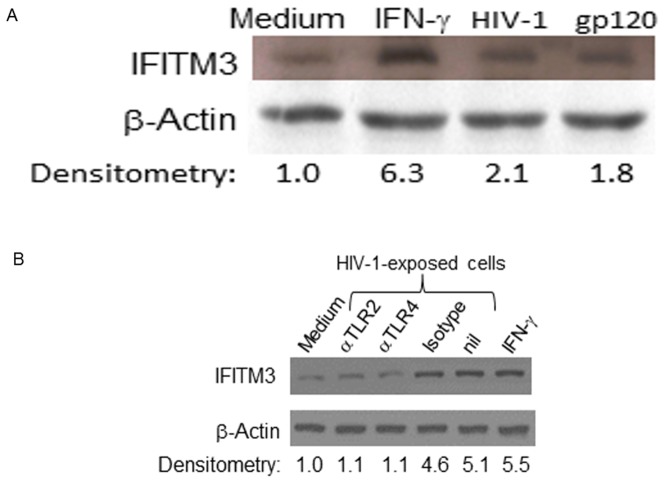
The exposure to HIV-1 or HIV-1 gp120 increases the IFITM3 level in epithelial cells. HeLa cells were exposed to HIV-1 (10 ng/mL p24 Ag), HIV-1 gp120 (5 µg/mL) or IFN-γ (10 ng/mL, used as a positive control) for 24 h. Cell monolayers were lysed with Laemmli buffer and submitted to blotting assays for IFITM3 protein, as described in Material and Methods. Bands were analyzed by densitometry using the ImageJ software 1.6.0, and the blotting is a representative figure of 3 assays. The basal levels of IFITM3 protein is shown in HeLa cells exposed only to culture medium.

We next measured the levels of IFITM3 in HeLa cells exposed to HIV-1, HIV-1 gp120 or IFN-γ, and found that IFITM3 levels doubled, when cells were exposed to HIV-1 or HIV-1 gp120, while IFN-γ enhanced IFITM3 levels around six times (Figure 3A). Consistently, HIV-1-induced enhancement of IFITM3 levels was prevented by the treatments with either anti-TLR 2 or 4 antibodies (Figure 3B).

**Figure 4:**
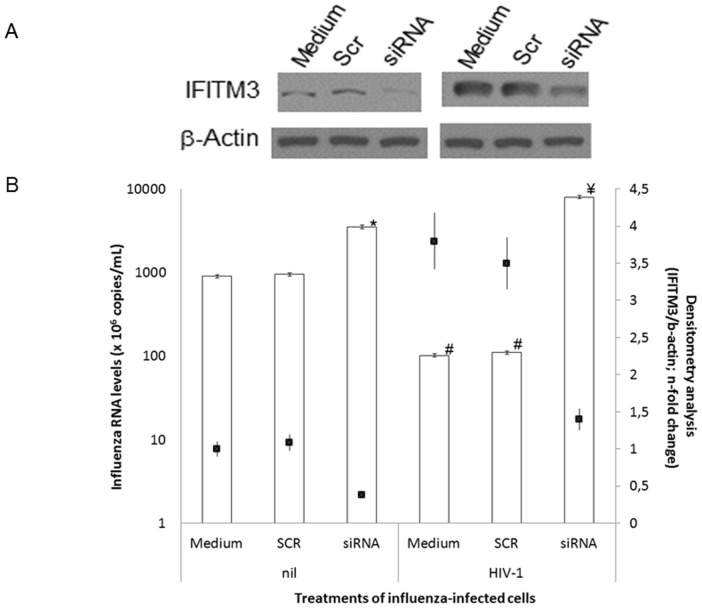
The HIV-1 inhibitory effect on A(H1N1)pdm09 replication is dependent on the IFITM3 expression. HeLa cells were transfected with 0.4(Scr) using lipofectamine 2000 and, after 6 h, cells were infected by A(H1N1)pdm09 (MOI 5) for 1 h. After that, cells were exposed to HIV-1 (10 ng/mL p24 Ag) for 24 h after influenza infection. (**A**) Monolayers were lysed with Laemmli buffer and submitted to blotting assay for the proteins IFITM3 and β-actin, as an housekeeping. Top panel: cells not exposed to HIV-1, and bottom panel: cells exposed to HIV-1. (**B**) The A(H1N1)pdm09 levels in culture supernatants were quantified by qRT-PCR (y-axis) and the immunoblotting bands were analyzed by densitometry using the ImageJ software 1.6.0 and n-fold changes of AU is scored as black squares (z-axis). Panel A is representative of three independent experiments. ^*^ *P*<0.05 for influenza replication in siRNA vs. medium (or Scr) treated cells, not exposed to HIV-1. ^#^ *P*<0.05 for influenza replication in HIV-1-exposed vs. non-exposed cells (medium or Scr). ^¥^ *P*<0.05 for influenza replication in siRNA vs. medium (or Scr) treated cells, exposed to HIV-1.

To confirm IFITM3 participation in the HIV-1-induced inhibition of influenza replication, we performed IFITM3 knocked down assays. HeLa cells transfected with siRNA for IFITM3 (or with its scrambled control; Scr) were infected with A(H1N1)pdm09, exposed to HIV-1 or culture medium, and, 24 h after influenza infection, cell supernatants and monolayers were harvested to quantify influenza and IFITM3 levels, respectively. Figure 4A shows that IFITM3 was knocked down from 60 to 50%, both in the absence (top panel) and presence (bottom panel) of HIV-1, preventing the increment in IFITM3 levels induced by this retrovirus. Of note, HIV-1-induced ability to enhance IFITM3 levels is reinforced in Figure 4A (comparing top and bottom panels). Moreover, the knocking down of IFITM3 led to an increase in influenza A(H1N1)pdm09 replication by three times (Figure 4B). In parallel to HIV-1-triggered IFITM3 enhancement (Figure 4B), influenza A(H1N1)pdm09 replication dropped by 88% (Figure 4B). As hypothesized, knockdown of IFITM3, prevented HIV-1's ability to inhibit influenza A(H1N1)pdm09 replication (Figure 4B).

### A(H1N1)pdm09 life cycle is restricted in HIV-1-infected macrophages

Although influenza does not undergo a productive life cycle in macrophages, it is able to adsorb onto, penetrate into and produce some viral proteins in these cells [7], [50]. Once infected by influenza, macrophages could orchestrate the host immune response and/or produce pro-inflammatory cytokines. Thus, we subsequently used human monocyte-derived macrophages to investigate whether HIV-1 infection could change the dynamics of influenza A(H1N1)pdm09 life cycle. To this end, macrophages were inoculated with A(H1N1)pdm09 at different MOIs and evidence of productive replication was searched daily. No signs of infectious influenza progeny were observed at either high or low MOIs (Figure S6). In agreement with the non-permissiveness of macrophages upon influenza infection [51], [52], no productive viral replication was detected in our assays with these cells.

**Figure 5:**
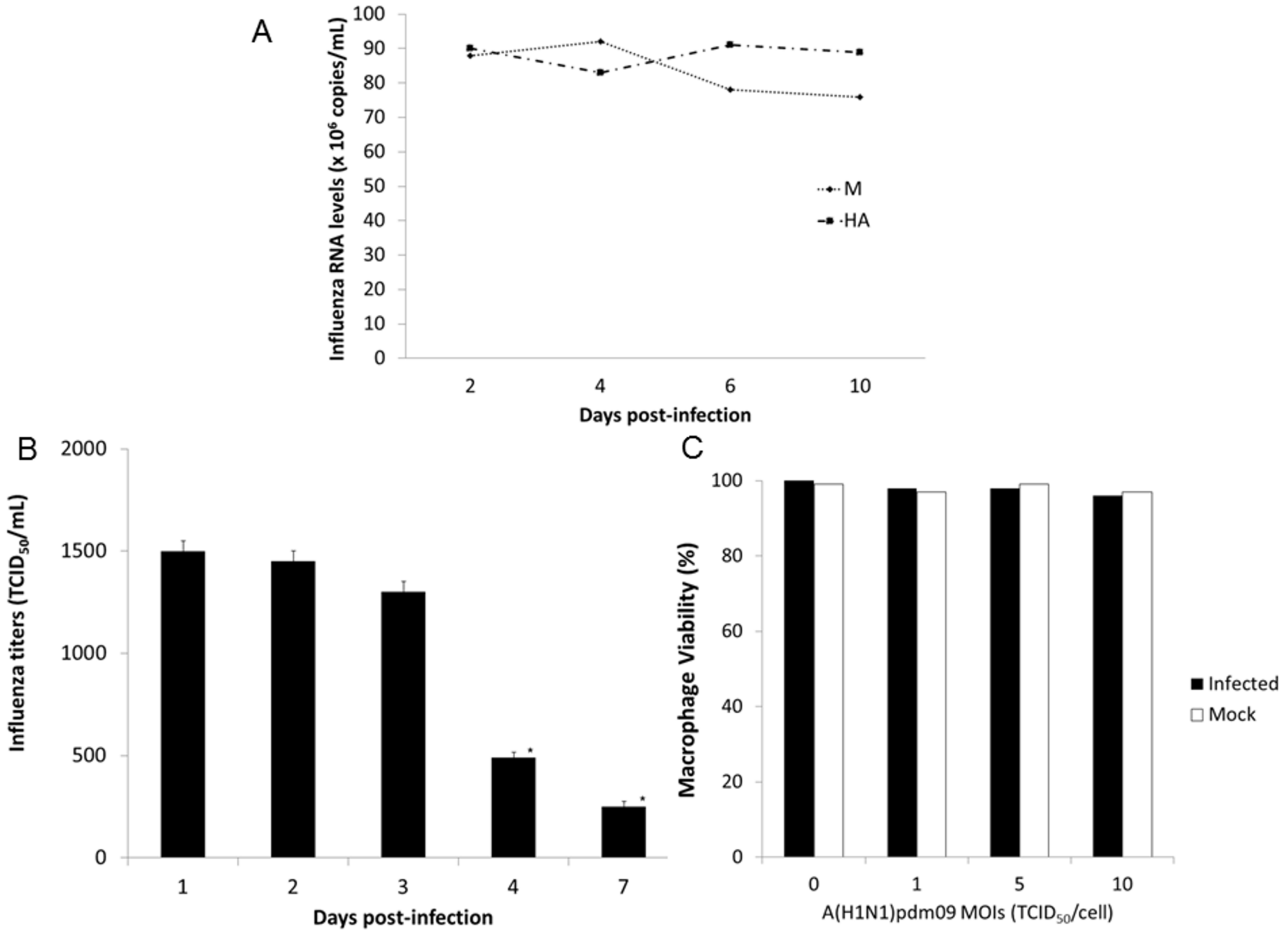
Recovery of Influenza from macrophages exposed to A(H1N1)pdm09. (**A**) Macrophages were exposed to A(H1N1)pdm09 (MOI  = 5) and, at different days after exposure, the influenza virus was quantified by qRT-PCR for M1 and HA genes in the cell culture supernatant (n = 3). (**B**) MDCK cells were infected with culture supernatant collected at different times from A(H1N1)pdm09-exposed macrophages (MOI  = 5) and influenza was tittered [36] (n = 3). (**C**) Macrophages were exposed to MOIs 1, 5 or 10 MOI of A(H1N1)pdm09, or to mock infection, and the cell viability was assessed by the XTT technique three days after influenza exposure (n = 3).

Therefore, a different approach was used, macrophages were infected with influenza (MOI  = 5) and the inoculum was not removed. Instead, at different times, the supernatant of the cultures were collected and the remaining amounts of A(H1N1)pdm09 measured. No increment in the influenza amounts was observed, as detected by evaluating the levels of viral RNA in the culture supernatants (Figure 5A) and by titration in MDCKs (Figure 5B). As a matter of fact, the infectivity of the original inoculum dropped substantially after 3 days p.i. (Figure 4B). Of note, neither the influenza inoculum nor the conditioned medium of MDCKs (mock-infected medium) were cytotoxic for the macrophages (Figure 5C).

**Figure 6:**
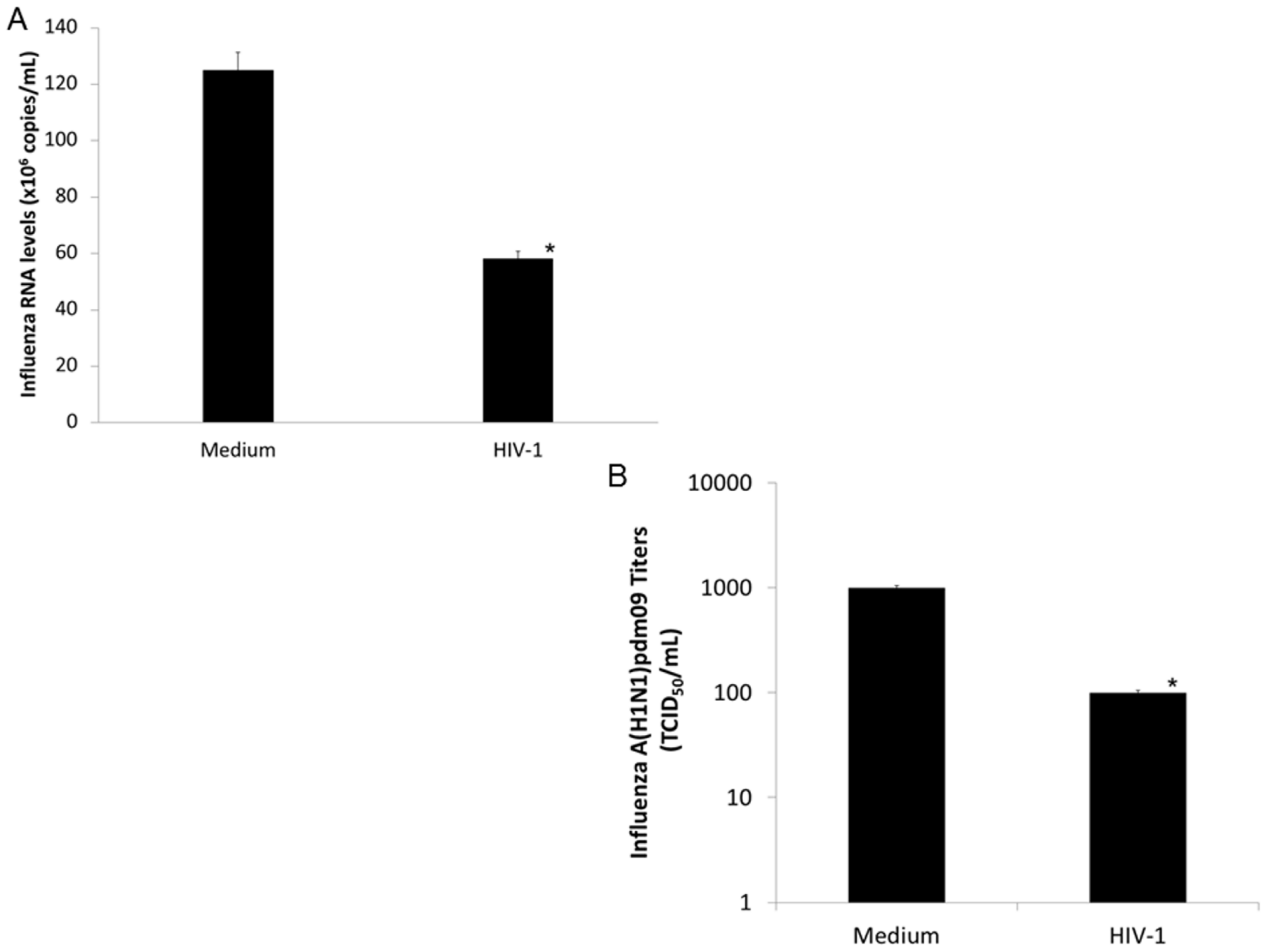
The HIV-1 infection reduces the A(H1N1)pdm09 level and infectivity. (**A**) HIV-1-infected macrophages (10 days of infection) were exposed to A(H1N1)pdm09 (MOI 5) for 3 days and, then, the influenza virus was titrated in the cell supernatant by qRT-PCR (n = 8). (**B**) Supernatants of similar cultures were added to MDCK cultures to evaluate the influenza infectivity after 72 h [36] (n = 3).

Next, considering that in primary macrophages the HIV-1 replication peaks around 15 days and that influenza infectivity inoculum remains constant for up to three days after exposure to these cells, we performed HIV-1/influenza co-infection assays in macrophages. HIV-1-infected macrophages (with 10 days of infection) were exposed to A(H1N1)pdm09 (MOI  = 5) for 3 days, and the amount and infectivity of influenza recovered from the supernatants were monitored. We found that A(H1N1)pdm09 RNA levels (Figure 6A) and infectivity in MDCKs (Figure 6B) were decreased by 40% and 90%, respectively, in the supernatant of HIV-1-infected macrophages. These results were consistently reproduced in macrophages from eight different healthy donors. Although macrophages are the only human cell type that could be infect by both HIV-1 and influenza virus simultaneously, absence of A(H1N1)pdm09 productive infection in these cells may limit further mechanistic analyses. Nevertheless, because influenza virus has shown a diminished infectivity in the presence of HIV-1, our findings seem to be consistent with the mild clinical outcomes of HIV-1/A(H1N1)pdm09 co-infected individuals [5], [25], [26], [29], [53].

### HIV-1 increases IFITM3 content and tolls influenza life cycle in human primary macrophages

**Figure 7:**
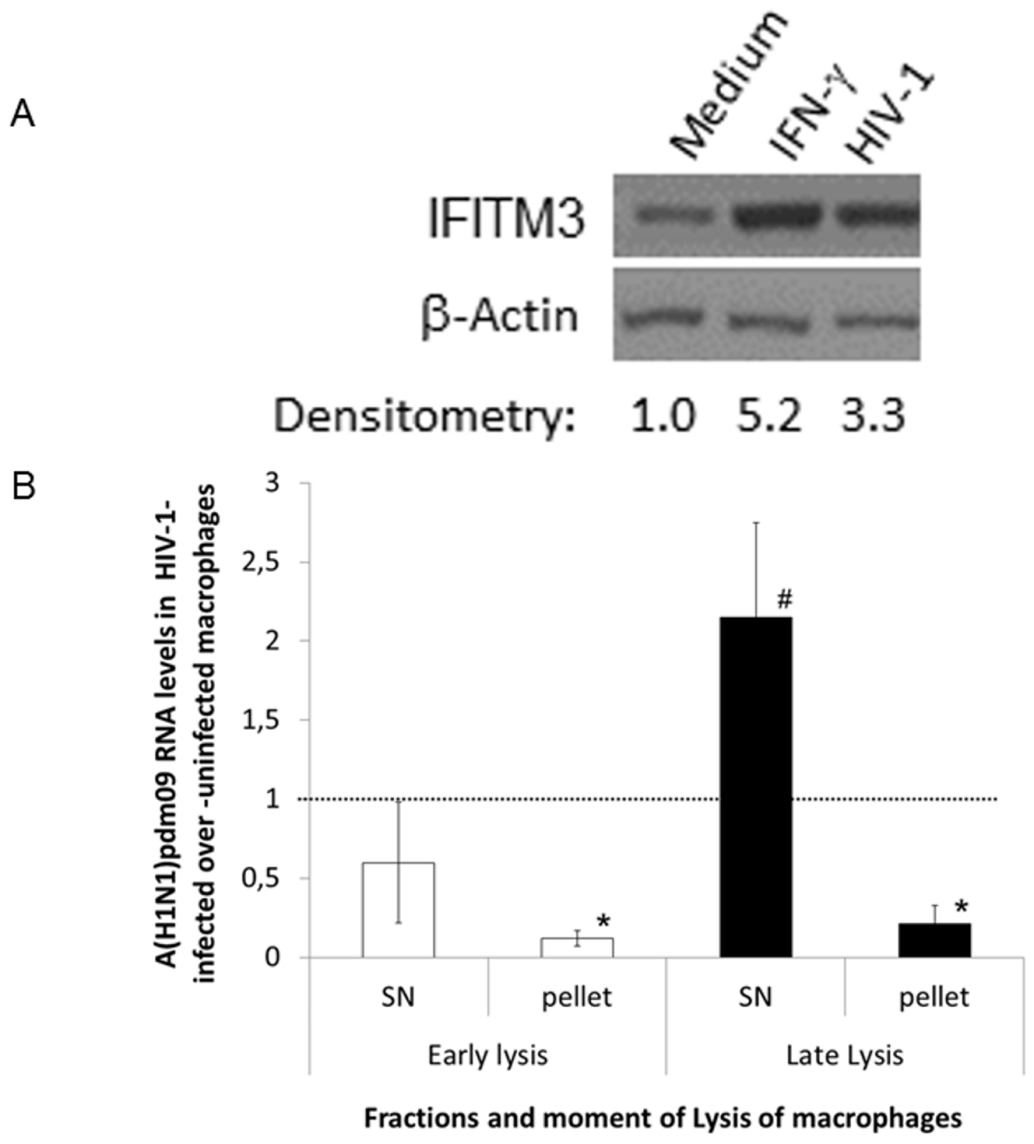
The increase of IFITM3 level in macrophages reduces A(H1N1)pdm09 penetration in *in vitro* co-infection. (**A**) HIV-1-infected macrophages (with 10 days of infection) were lysed with Laemmli buffer and the lysate was subjected to blotting assays for IFITM3 protein. The bands, representative of three different assays, were analyzed by densitometry using the ImageJ software 1.6.0. and arbitrary units scored. (**B**) In parallel, macrophages infected or not by HIV-1 were infected by A(H1N1)pdm09 (MOI 5) for 1 h at 4°C. To analyze adsorption, cells were washed just after the incubation at 4°C and lysed with buffer A. To analyze penetration, cells were washed after 1 h at 4°C, the monolayers were covered with medium and the temperature was raised to 37°C for 6 h, when buffer A was added. Alternatively, temperature was kept at 37°C for 24 h, when buffer A was added. Lysates were fractioned to nuclear and non-nuclear fractions by centrifugation. A(H1N1)pdm09 RNA was extracted from these fractions and quantified by qRT-PCR (relative quantification). ^*^ *P*<0·05 for decrease in influenza A(H1N1)pdm09 RNA levels in HIV-1- vs. mock-infected macrophages. ^#^ *P*<0·05 for increase in influenza A(H1N1)pdm09 RNA levels in HIV-1- vs. mock-infected macrophages.

We subsequently evaluated whether HIV-1 infection also enhances IFITM3 in human primary macrophages. The HIV-1 infection augmented by three times the macrophage IFITM3 levels (as detected 14 days after infection), relative to IFITM3 contents in uninfected cells (Fig. 7A). Of note, we tried to perform siRNA assays to knockdown IFITM3 in macrophages; however, knockdown efficiency varied severely from donor to donor, precluding further mechanistic analyzes based on this strategy. Nevertheless, as it has been shown that IFITM3 restricts influenza life cycle by preventing arrival of viral ribonucleoproteins (RNPs) to the host cell nucleus [54], we measured the levels of A(H1N1pdm)09 bound to plasma membrane and the amount of viral RNPs that reached the nucleus of HIV-1-infected macrophages at early and late time points, by synchronizing influenza infection and measuring influenza RNA levels in the macrophage nuclear and non-nuclear fractions (see Material and Methods for details). Consistently with the mechanism of action of IFITM3, A(H1N1)pdm09 penetration into the nucleus was reduced by 80%, in HIV-1-infected macrophages (Figure 7B; pellet fraction from the early lysis). No significant changes in A(H1N1)pdm09 adsorption was observed in HIV-1-infected macrophages (Figure 7B; supernatant fraction from the early lysis). At a later time point (24h after A(H1N1)pdm09 addition to HIV-1-infected macrophages), increased levels of influenza RNA was detected in the non-nuclear cell fraction (Figure 7B; supernatant from the late lysis), concomitant with the low amount of influenza RNA found in the nuclear fraction (Figure 7B; pellet from the late lysis). The increment in influenza levels in the non-nuclear fraction was consistent with the diminishment of this virus infectivity in the presence of HIV-1, as measured by titration in MDCKs (Figure 6). It is reasonable to imagine that in the presence of HIV-1, A(H1N1)pdm09 virus particles would enter into the macrophages, but their way to the nucleus would be impaired, limiting later steps of influenza life cycle.

**Figure 8:**
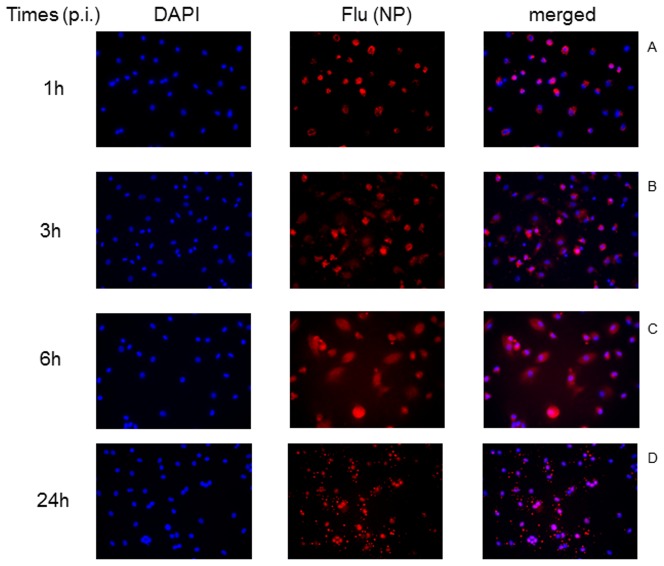
Dynamics of influenza infection in human primary macrophages. Macrophages were infected with influenza for 1 (A), 3 (B), 6 (C) and 24h (D). Then, these cells were fixed with 4% paraformaldehyde and stained with anti-influenza NP (red) and the DNA probe DAPI (blue). Magnification 400 x.

**Figure 9:**
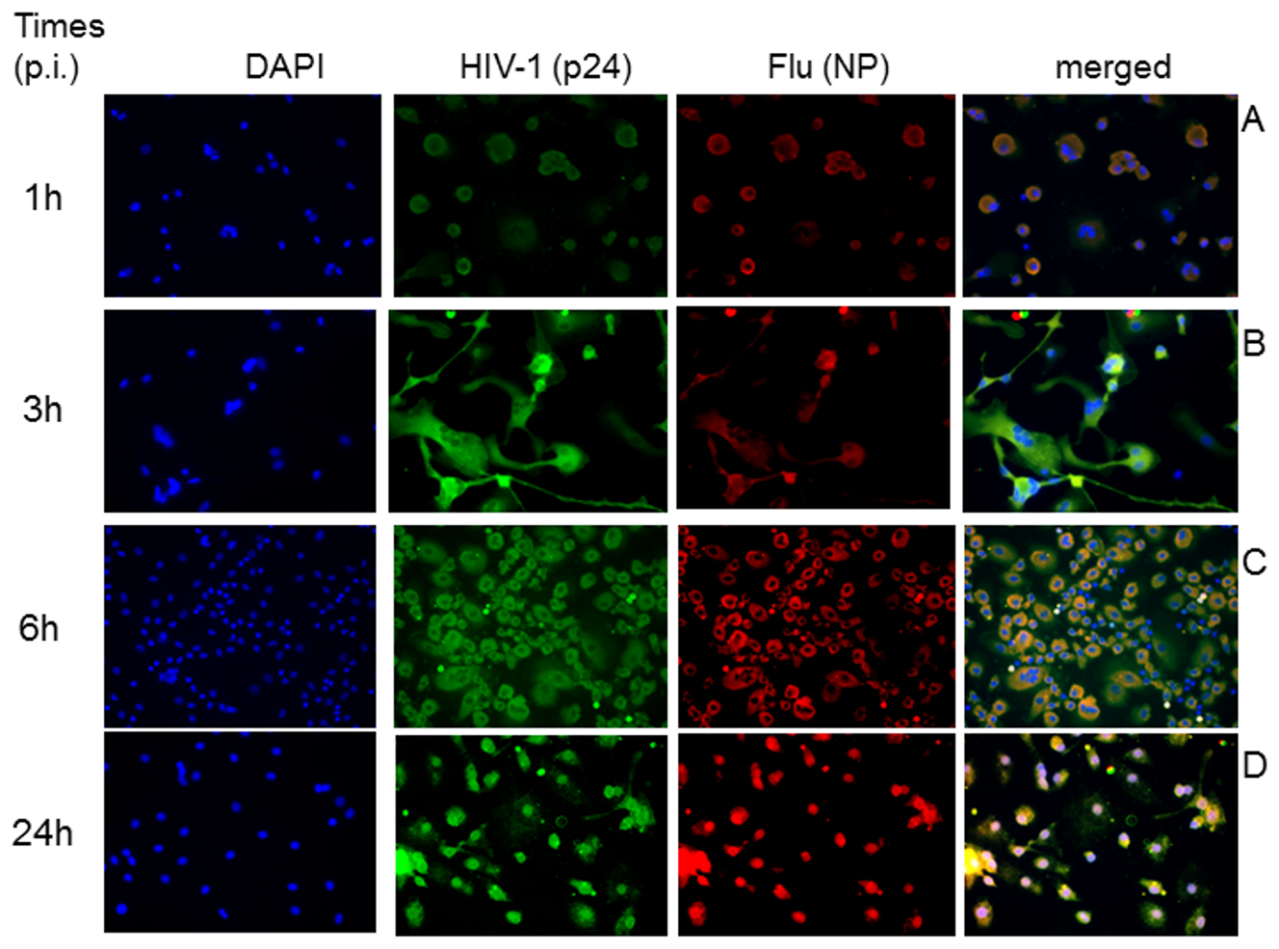
Dynamics of influenza infection in HIV-1-infected human primary macrophages. HIV-1-infected macrophages were infected with influenza for 1 (A), 3 (B), 6 (C) and 24 (D). Then, these cells were fixed with 4% paraformaldehyde and stained with anti-HIV-1 p24 (green), anti-influenza NP (red) and DNA probe DAPI (blue). Magnification 400 x.

To confirm these results and our interpretation on these quantitative analysis, we compared the A(H1N1)pdm09 distribution in the nuclei of HIV-1-infected and uninfected macrophages. Influenza infection in macrophages in the absence and presence of HIV-1 is shown in Figures 8 and 9, respectively. With respect to macrophages infected with A(H1N1)pdm09 only, NP localizes superficially in most of the cells, while some already display this protein in their nuclei, at 1 h after infection (Figure 8A). Subsequently, NP nuclear distribution progressively increases at 3 and 6 h after infection (Figure 8B and C). At late time points, nuclear localization of NP is more intense (Figure 8D). With respect to influenza co-infection in HIV-1-infected macrophages, no NP signals are found within the nuclei at early time points (Figure 9A to C). At 24 h after influenza infection, NP is observed in the nuclei of HIV-1-infected macrophages (Figure 9D), but also diffused throughout the cytoplasm – differently than what has been observed in macrophages infected only with influenza (Figure 8D). Altogether, our data (from Figures 7 to 9) suggests that influenza RNP progress to the nuclei of HIV-1-infected macrophages is impaired/delayed.

**Figure 10:**
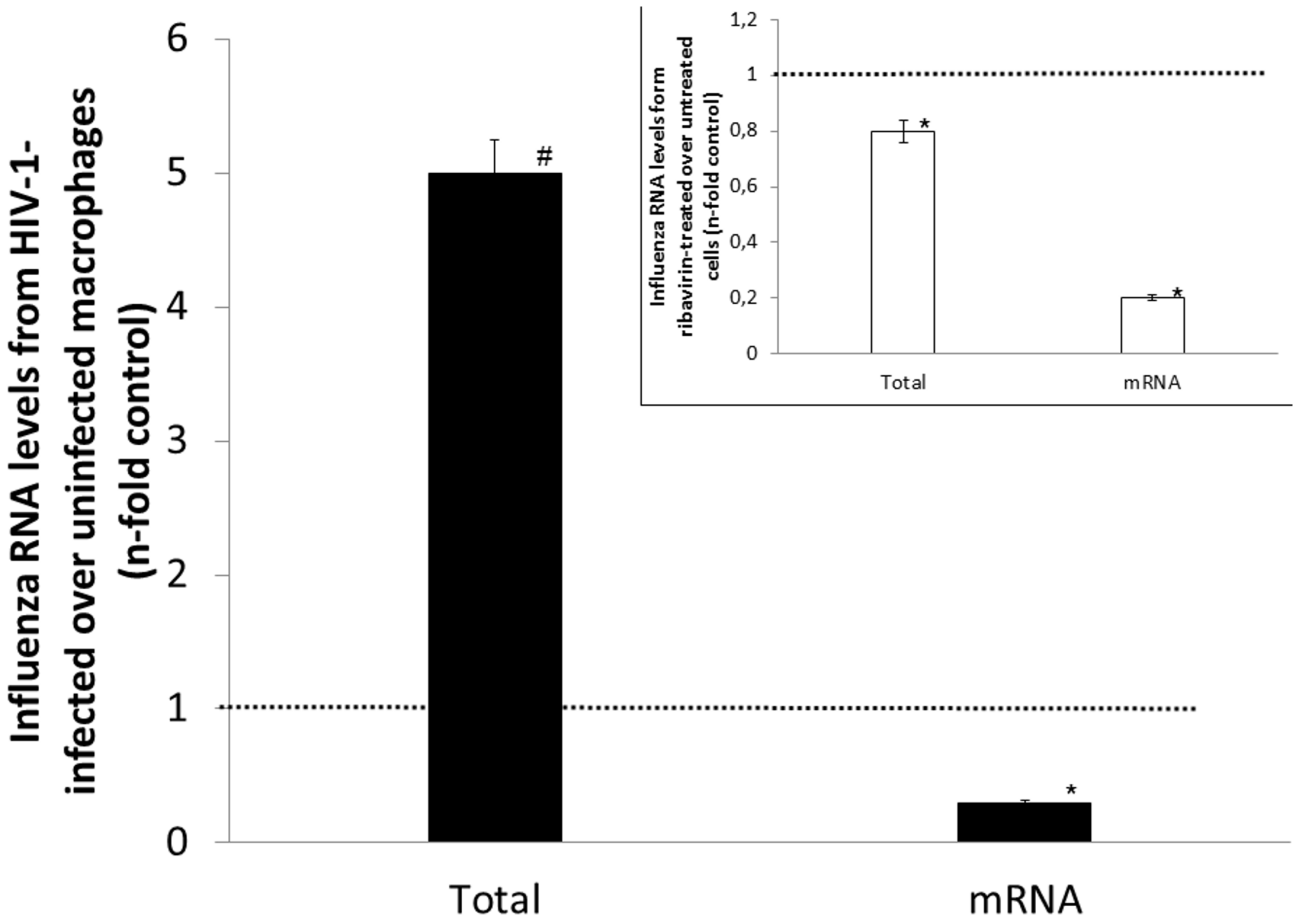
Measurements of influenza transcription. HIV-1-infected and -uninfected macrophages were infected with influenza A(H1N1)pdm09. After 24h, cells were lysed, RNA extracted and cDNA synthetized using oligo.dT and UNI-12 primers. Quantitative real time PCR was performed for the M1 gene. As a control, ribavirin-treated and -untreated influenza-infected macrophages were submitted to the same procedure described above (inset). * *P<*0.05 for reduction of RNA levels. ^#^ *P*<0.05 for increase of RNA levels. (n = 6).

Considering that, we analyzed the total A(H1N1)pdm09 RNA and mRNA in HIV-1-infected over -uninfected macrophages. Total influenza RNA levels are increased in HIV-1-infected macrophages, despite their reduced ability to reach the nuclei (Figure 10). Naturally in the present context, influenza RNA transcription is reduced in HIV-1-infected cells (Figure 10). As a control, ribavirin was used to inhibit mainly influenza transcription (Figure 10; inset) [55].

**Figure 11:**
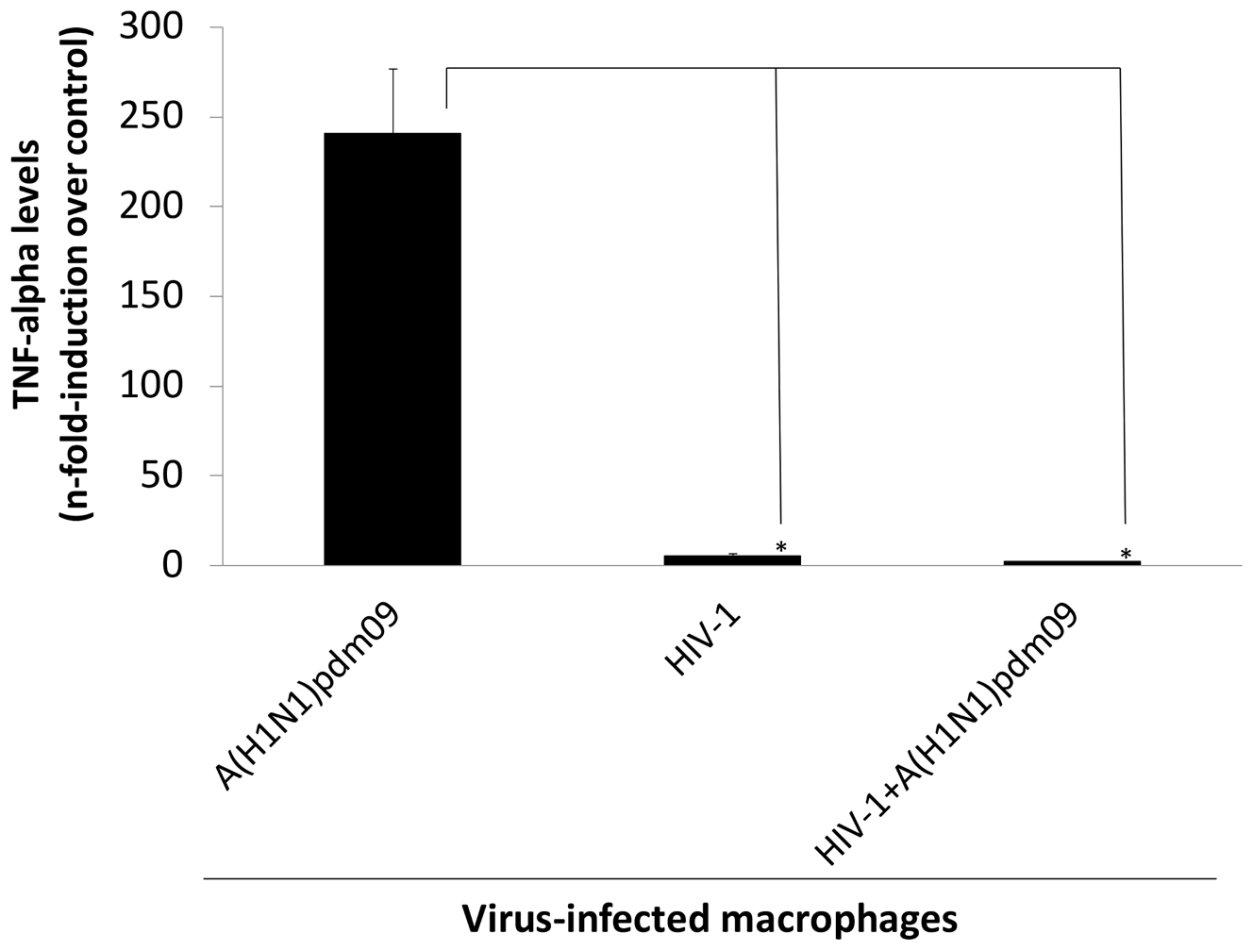
Influenza A(H1N1)pdm09 induction of TNF-α is attenuated in HIV-1-infected macrophages. Macrophages, infected or not with HIV-1, were co-infected by A(H1N1)pdm09 for 1 h at 37°C. Cells were than washed and TNF-α levels were measured 24 h after influenza infection. * *P*<0.05 for decrease TNF-α levels in macrophages co-infected or infected by HIV-1 vs. influenza-infected macrophages.

We subsequently wondered whether due to HIV-1-imposed early restriction on influenza life cycle in macrophages, the ability of this orthomyxovirus to induce the production of pro-inflamatory cytokines, such as TNF-α, would be impaired. Macrophages, infected by HIV-1, were exposed to influenza A(H1N1)pdm09 and the levels of TNF-α were measured. As we can see (Figure 11), the influenza A(H1N1)pdm09's ability to enhance the TNF-α levels is reduced in HIV-1-infected macrophages. Therefore, HIV-1-induced enhancement in IFITM3 levels in macrophages may not only restrict influenza life cycle in this cell, but could also prevent influenza-induced pro-inflamatory cytokine release.

### Influenza A(H1N1)pdm09 evolution is delayed in the presence of HIV-1 in *in vitro* experiments and co-infected individuals

We investigated whether in NPAs from HIV-1-infected individuals with positive diagnosis for influenza A(H1N1)pdm09 both viruses could be detected. If so, this would suggest the presence of these agents at the same anatomical site. Indeed, we found these viruses genome in 5 out of 7 respiratory samples of co-infected patients, displayed in Table S1.

**Figure 12:**
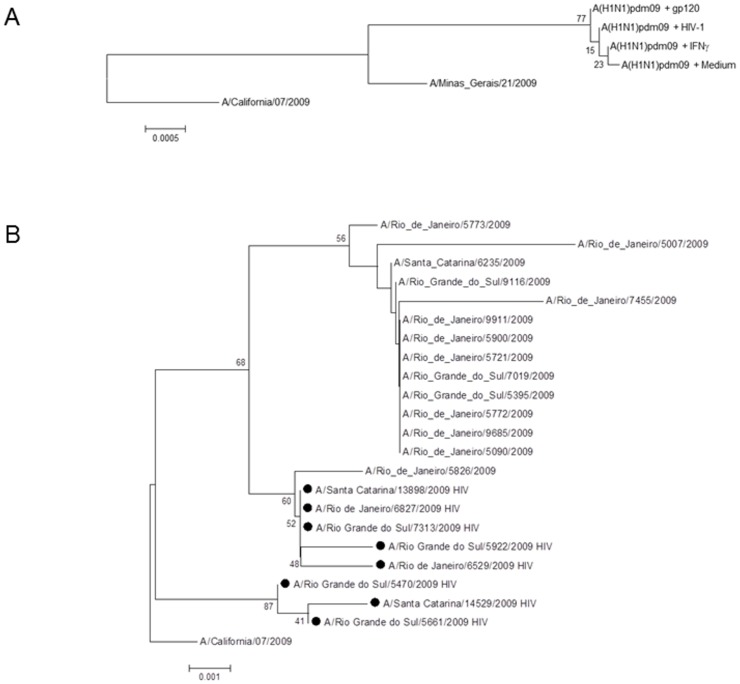
Molecular analysis of A(H1N1)pdm09 HA gene. (**A**) After 2 h of A(H1N1)pdm09 infection (MOI  = 5), HeLa cells were exposed to culture medium or treatment with HIV-1 (10 ng/mL p24 Ag), gp120 (5 µg/mL) or IFN-γ (10 ng/mL). After 24 h of influenza infection, the supernatant was harvested, RNA was extracted, the HA gene of influenza was sequenced and presumptive amino acid residues analyzed. (**B**) Clinical samples from influenza A(H1N1)pdm09-infected patients co-infected or not with HIV-1 with onset of illness detected during July 18^th^ to August 13^th^, 2009 at Southern Brazil and Rio de Janeiro had their RNA extracted, nucleotide were sequenced and putative amino acid sequences for HA analyzed. Trees are rooted by vaccinal strain A/California/07/2009. Circles indicate samples from co-infected individuals. Sequencing of HA was performed by the Sanger method and the phylogenetic tree of amino acids generated in the program Mega 5.2, with the Neighbor-joining algorithm and 2000 of bootstrap. The bootstrap probability is indicated for each interior branch. The scale bar indicates the number of amino acid changes per site.

This co-detection around 70% suggests that HIV-1 could influence influenza evolution. We analyzed the influenza genome from both co-infection experiments and patients. We sequenced the A(H1N1)pdm09 hemagglutinin (HA) gene that escaped from HIV-1-, gp120- and IFN-γ-induced inhibition. We chose for sequencing this gene, because it has the highest evolution rate amongst influenza genes. As experimental infection assays were performed in quadruplicate and produced similar HA sequences, representative consensus are displayed in the phylogenetic tree and compared with parental strains (Figure 12A). The analysis of the translated nucleotide sequences reveals that evolution of A(H1N1)pdm09 virus was slower in the presence of HIV-1, gp120 or IFN-γ than that observed for virus cultured in the presence of medium only (Figure 12A), highlighting that HIV-1-induced inhibition of A(H1N1)pdm09 replication resulted in an impaired influenza evolution. Of note, the differences in virus evolution among the experimental groups are very small, since the presence of cell-culture-derived changes, P199Q and Q240R in comparison to the parental viruses (A/California/07/2009, vaccinal strain, and A/Minas Gerais/490/2009, primary isolate), forced the sequences to cluster together.

We next analyzed HA sequences of A(H1N1)pdm09 viruses collected from HIV-1-infected individuals (Table S2) and compared to A(H1N1)pdm09 specimens collected from HIV-1 seronegative individuals at the same period (from July 18^th^ to August 13^th^, 2009) and geographical region (Southern Brazil and Rio de Janeiro). As we can see in Figure 12B, A(H1N1)pdm09 virus from co-infected individuals (Figure 12B, circles) are phylogenetically closer to vaccinal strain/index case of 2009 pandemics, A/California/07/2009, than to most of other samples from the same period and region. Evolution of A(H1N1)pdm09 viruses from HIV-1-infected individuals seems to have being impaired when compared to other samples from similar geographical region and time period (Figure 12B). Consequently, no specific pattern of mutations existed to cluster A(H1N1)pdm09 sequences from HIV-1 co-infected individuals together (Figure 12B, circles), with the exception of a small cluster from influenza sequences of southern Brazil (Figure 12B, see influenza strains A/Rio Grande do Sul/5470/2009, A/Rio Grande do Sul/5661/2009 and A/Santa Catarina/14529/2009). Regarding specific mutations, when HA sequences from co-infected individuals were compared with others from mild to fatal cases (81 specimens), it is possible to observe that Brazilian strains have the changes P100S, I338V and Q310R, while the mentioned three strains from southern Brazil have only Q310R and S220T changes. The reduced influenza evolution in the presence of HIV-1 may reflect a compromised A(H1N1)pdm09 replication and may correlate with mild clinical outcomes for co-infected individuals living in different regions of the world [25].

## Discussion

Increased worldwide mortality was observed during the 2009 Influenza pandemics for especial groups of patients at higher risk, such as individuals immunocompromised [23], [24] by cancer or transplantations [24]. Surprisingly, HIV-1-infected individuals presented a benign clinical outcome when infected by A(H1N1)pdm09 [25]–[30], unless when in AIDS [29]. Although some empirical explanations have been raised to clarify this phenomenon, such as anti-A(H1N1)pdm09 activity of antiretroviral drugs or enhanced medical attention paid to these patients, the possibility that the concomitant infection with HIV-1 may attenuate the replication of Influenza virus and, thus, mitigate the infection clinical course has not been systematically evaluated. Since it has been reported that HIV-1 infection may lead the host to a new homeostatic equilibrium and confer resistance to some co-pathogens [16], we evaluated in this study whether HIV-1 infection could interfere in influenza A(H1N1)pdm09 life cycle. This study represents a challenging approach, because these agents primarily infect different cell types. Nevertheless, relevant insights made here may contribute to understand better the outcome of the clinical episodes of HIV-1/influenza co-infection.

In brief, HIV-1 or its gp120 inhibits A(H1N1)pdm09 replication in epithelial cells in an IFITM3-depedent fashion in primary macrophages and epithelial cells. As a consequence of decreased A(H1N1)pdm09 replication in the presence of HIV-1, an impaired influenza evolution was observed in the *in vitro* experiments and in co-infected patients. Our findings may correlate with the mild clinical course of influenza infection found in co-infected individuals.

Since the respiratory tract of HIV-1-infeccted individuals may be colonized by opportunistic, and indeed rare, pathogens, such as *Pneumocystis carinii* and *P. jiroveci* – this anatomical site is hardly think as able to albergate HIV-1and influenza co-infection. Here, we also showed both viruses were found in respiratory secretions from co-infected hosts. Considering that influenza is a seasonal virus, it may infect HIV-1-postive patients many times throughout the life-span of these individuals. Therefore, HIV-1 replication may be influenced and also influence influenza life cycle. HIV-1 replicates in alveolar macrophages [21], [22], and the respiratory tract may constitute a viral sanctuary, since antiretroviral resistance mutations may be found in the sputum of individuals with undetectable, HAART-suppressed, viremia [21], [22]. Besides, influenza infection triggers a pulmonary inflammatory response with subsequent homing of monocytes to the respiratory tract [5], [57], thus increasing the number of HIV-1 permissive cells. In HIV-1/influenza co-infected individuals, some of these CD4+ cells homing to the respiratory tract due to influenza infection may also harbor latent proviral HIV-1 genome in their nucleus; thus, upon activation they may actively produce the mentioned retrovirus – which could potentially increase HIV-1 viral loads in the respiratory tract. A(H1N1)pdm09 active replication in the respiratory tract occurs in epithelial cells, and also in macrophages [57]. Despite that, influenza-infected macrophages progeny is uninfectious, as shown here and elsewhere [51].

HIV-1 is unable to enter within epithelial cells, but viral particles or its soluble glycoproteins may interact with their surface, via TLR 2 and 4 [13] – which could trigger an IFN dependent response [12], culminating with the increment in IFITM3 levels. Therefore, influenza-infected cells may be continuously exposed to HIV-1 viral particles and to HIV-1 surface protein gp120 in the respiratory tract of HIV-1/A(H1N1)pdm09 co-infected patients. Thus, we reasoned that evaluating the impact on influenza replication resulting from A(H1N1)pdm9-infected epithelial cells exposed to HIV-1 or gp120 could constitute a valuable model to get insights on the relatively benign clinical evolution of influenza infection in HIV-1-infected patients. Here, we found that HIV-1 and gp120 produced a TLR2/4-dependent IFN-like effect towards influenza replication, revealed by the IFITM3-dependent HIV-1- or gp120-induced inhibition of influenza replication. The IFITM3 effect on influenza life cycle could toll influenza replication, decreasing viral evolution and, perhaps, contributing to the mild clinical symptoms observed in our groups of HIV-1/A(H1N1)pdm09 co-infected individuals.

We also used human primary macrophages in our experimental studies. Macrophages function as natural reservoirs of HIV-1 and sustain long lasting virus replication [15], whereas this cell is non-permissive for influenza [51]. That is, infectious influenza virus enters into macrophages, viral proteins are synthetized, but no infectious progeny are made [58]. Initially, macrophage susceptibility to influenza may allow this cell to act like a “Trojan horse”, by allowing antigen presentation via class I MHC and, therefore, orchestration of an cell-mediated cytotoxic antiviral response [10]. On the other hand, hospitalization due to influenza infection occurs due to symptoms of severe acute respiratory infection (SARI), which are associated with an enhanced pro-inflammatory response by influenza-infected macrophages [11], [59]–[62]. Indeed, we observed that A(H1N1)pdm09 virus titer dropped over time in inoculated human primary macrophages, supporting the notion that no infectious virus progeny is generated. After inoculation of HIV-1-infected macrophages with A(H1N1)pdm09, the infectivity of influenza virus recovered from culture supernatants is significantly smaller than in supernatants from macrophages not infected by HIV-1. We also found that HIV-1 enhances IFITM3 content in macrophages. As it has been proposed, IFITM3 inhibits influenza replication by impairing the arrival of viral RNPs into the nucleus [56]. In fact, we showed that in HIV-1-infected macrophages, A(H1N1)pdm09 RNA translocation into the nucleus was reduced/delayed and, consequently, influenza transcription in HIV-1-infected macrophages is attenuated. This phenomena may have reduced influenza-ability to induce TNF-α, which is one of the most important cytokines released during influenza-induced cytokine storm [51], [63]. Therefore, we imagined that chances of cytokine storm in HIV-1/influenza co-infected individuals would also be reduced, which could have contributed to the surprise mild clinical outcomes observed for this group of risk during the 2009 pandemics.

Our results, on HIV-1/influenza co-infection in macrophages could gain an even more substantial meaning if correlated with clinical-histological findings. The work from Shieh et al. shows representative immunohistochemistry results from individuals that deceased due to influenza infection [57]. They naturally observed influenza presence in macrophages of the respiratory tract [57]. Moreover, in their cohort, 4% of the individuals were HIV-1 positive, but no correlation with the existence of this retrovirus in the respiratory tract has been made. Therefore, to the best of our knowledge, clinical-histological investigation on influenza/HIV-1 co-infection in macrophages is aim of further investigation.

In conclusion, our collection of data, obtained from experimental assays and from clinical samples, brings new suggestions that HIV-1, through mature virions or its envelope protein, may reduce the replication and delay the evolution of A(H1N1)pdm09 virus, thus contributing to attenuate the clinical course of Influenza in HIV-1/A(H1N1)pdm09 co-infected patients.

## Supporting Information

Figure S1 **Purity of the RNA preparation from nuclear extracts.** Macrophages were lysed with buffer A and centrifuged for 10 min. at 1000× *g*. The obtained pellet and supernatant (SN) contain the nuclear and non-nuclear cell fractions, respectively. RNA from each of these fractions was extracted, cDNA synthetized and real time PCR performed. * *P*<0.001 for comparisons between corresponding white and black bars. Amplicon not detected (n.d.) whitin 45 cycles is indicated. (n = 6).
(TIF)

Figure S2 **The exposure to HIV-1 Tat has no effect on influenza A(H1N1)pdm09 replication.** A(H1N1)pdm09-infected HeLa cells were exposed to HIV-1 Tat, oxidized Tat (Tat-ox) or IFN-γ. After 24 h, the RNA was extracted from the culture supernatants, and the influenza virus was quantified by qRT-PCR (A) or supernatants were tittered in MDCKs (B). The IFN-γ was used as positive control. The asterisks indicate statistical significance (*P*<0.05) over control (medium). (n = 4).
(TIF)

Figure S3 **The exposure to HIV-1 inhibits influenza A(H1N1)pdm09 replication in a dose-dependent manner.** A(H1N1)pdm09-infected HeLa cells were exposed to indicated inputs of HIV-1 or IFN-γ. After 24 h, supernatants were tittered in MDCKs. The IFN-γ was used as positive control. The asterisks indicate statistical significance (*P*<0.05) over control (medium). (n = 3).
(TIF)

Figure S4 **Viability of HeLa cells exposed to HIV-1. HeLa cells were exposed to HIV-1 (10 ng/mL of HIV-1 p24 Ag), gp120 (5 µg/mL) or IFN-γ (10 ng/mL).** After 72 h, cell viability was measured by XTT assay. (n = 4).
(TIF)

Figure S5 **HIV-1 does not grow in HeLa cell lines.** The cell lineages HeLa and TZM-bl (which has the HeLa background but expresses CD4 and CCR5, among other genes) were exposed to HIV-1 (10 ng/mL of HIV-1 p24 Ag) for 1 h at 37°C. After that, cells were washed to remove unbounded viruses and fresh culture medium was added. At indicated days after infection, aliquots of the culture supernatants were collected to measure HIV-1 p24 Ag by ELISA. HIV-1 production was not detected (n.d.) in HeLa cells. (n = 4).
(TIF)

Figure S6 **Macrophages are non-permissive to influenza A(H1N1)pdm09.** Macrophages were infected with the indicated MOIs of influenza A(H1N1)pdm09 for 1 h at 37°C with inoculation medium (serum-free DMEM containing 0.2% serum albumin and trypsin at 4 µg/mL). After that, cells were washed to remove unbounded viruses and fresh inoculation medium was added. At indicated days after infection, aliquots of the culture supernatants were collected and tittered in MDCKs by end-point dilution (TCID_50_/mL). (n = 3).
(TIF)

Table S1 **Primer sequences.**
(DOCX)

Table S2 **Basic clinical information from HIV-1-infected individuals with laboratory-confirmed diagnosis of influenza A(H1N1)pdm09.**
(DOCX)
