# Supplementary figures and images for: HIV-1 and Its gp120 Inhibits the Influenza A(H1N1)pdm09 Life Cycle in an IFITM3-Dependent Fashion
Source: PLoS One. 2014 Jun 30;9(6):e101056. doi: 10.1371/journal.pone.0101056 (PMC4076258; doi:10.1371/journal.pone.0101056)

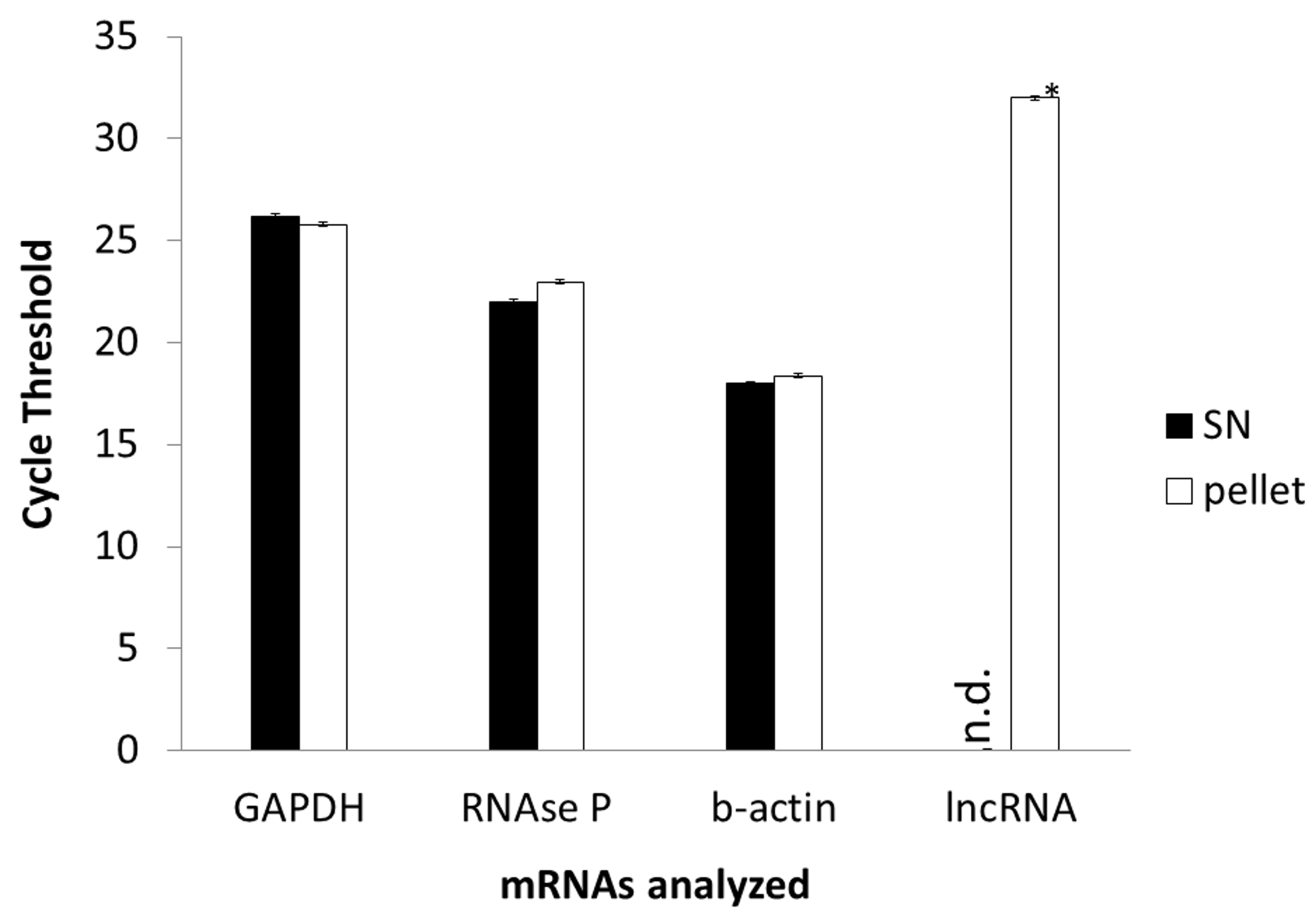

Supplement: Figure S1 — Purity of the RNA preparation from nuclear extracts. Macrophages were lysed with buffer A and centrifuged for 10 min. at 1000× g. The obtained pellet and supernatant (SN) contain the nuclear and non-nuclear cell fractions, respectively. RNA from each of these fractions was extracted, cDNA synthetized and real time PCR performed. * P<0.001 for comparisons between corresponding white and black bars. Amplicon not detected (n.d.) whitin 45 cycles is indicated. (n = 6). (TIF) [file pone.0101056.s001.tif]

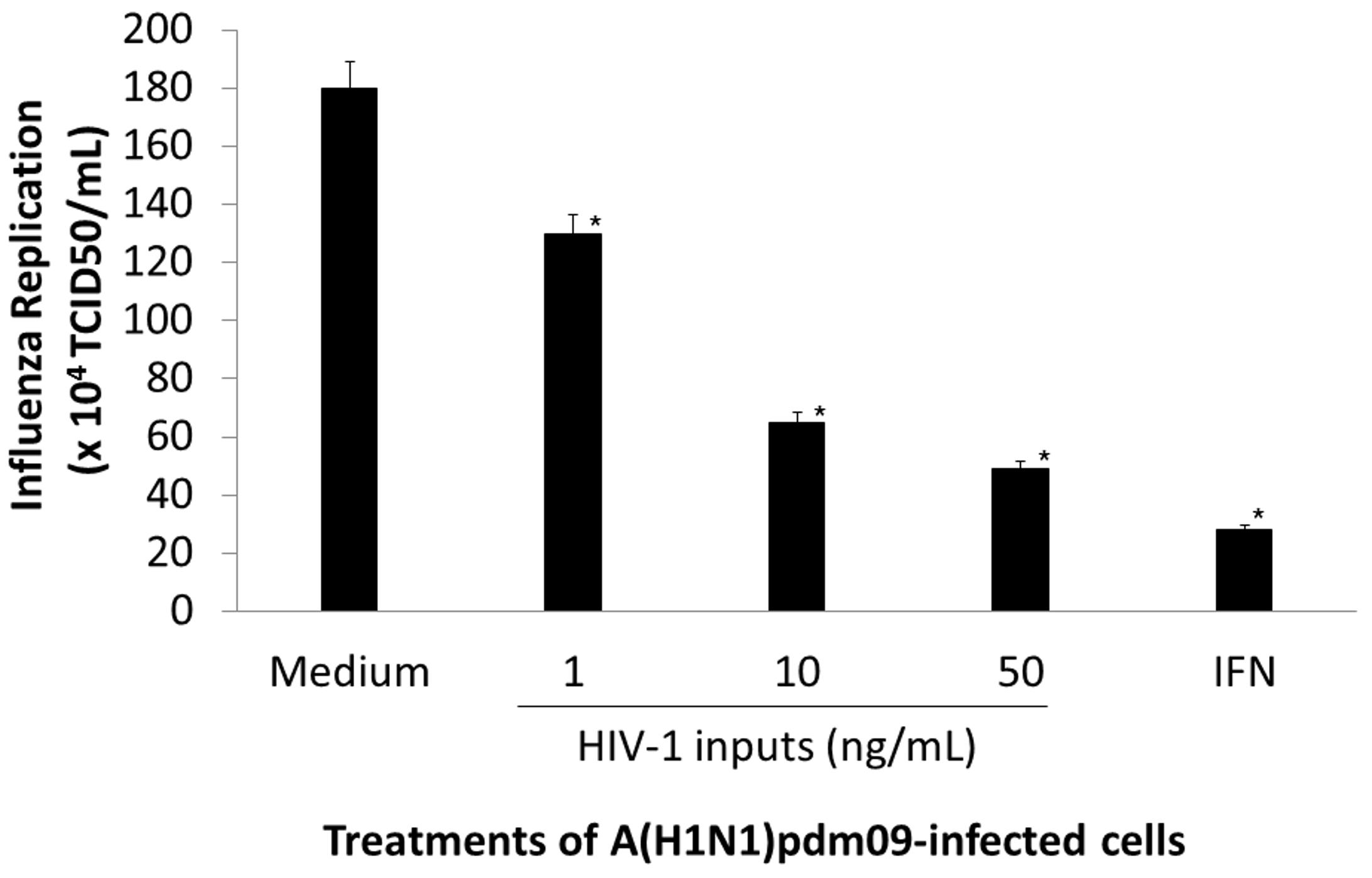

Supplement: Figure S3 — The exposure to HIV-1 inhibits influenza A(H1N1)pdm09 replication in a dose-dependent manner. A(H1N1)pdm09-infected HeLa cells were exposed to indicated inputs of HIV-1 or IFN-γ. After 24 h, supernatants were tittered in MDCKs. The IFN-γ was used as positive control. The asterisks indicate statistical significance (P<0.05) over control (medium). (n = 3). (TIF) [file pone.0101056.s003.tif]

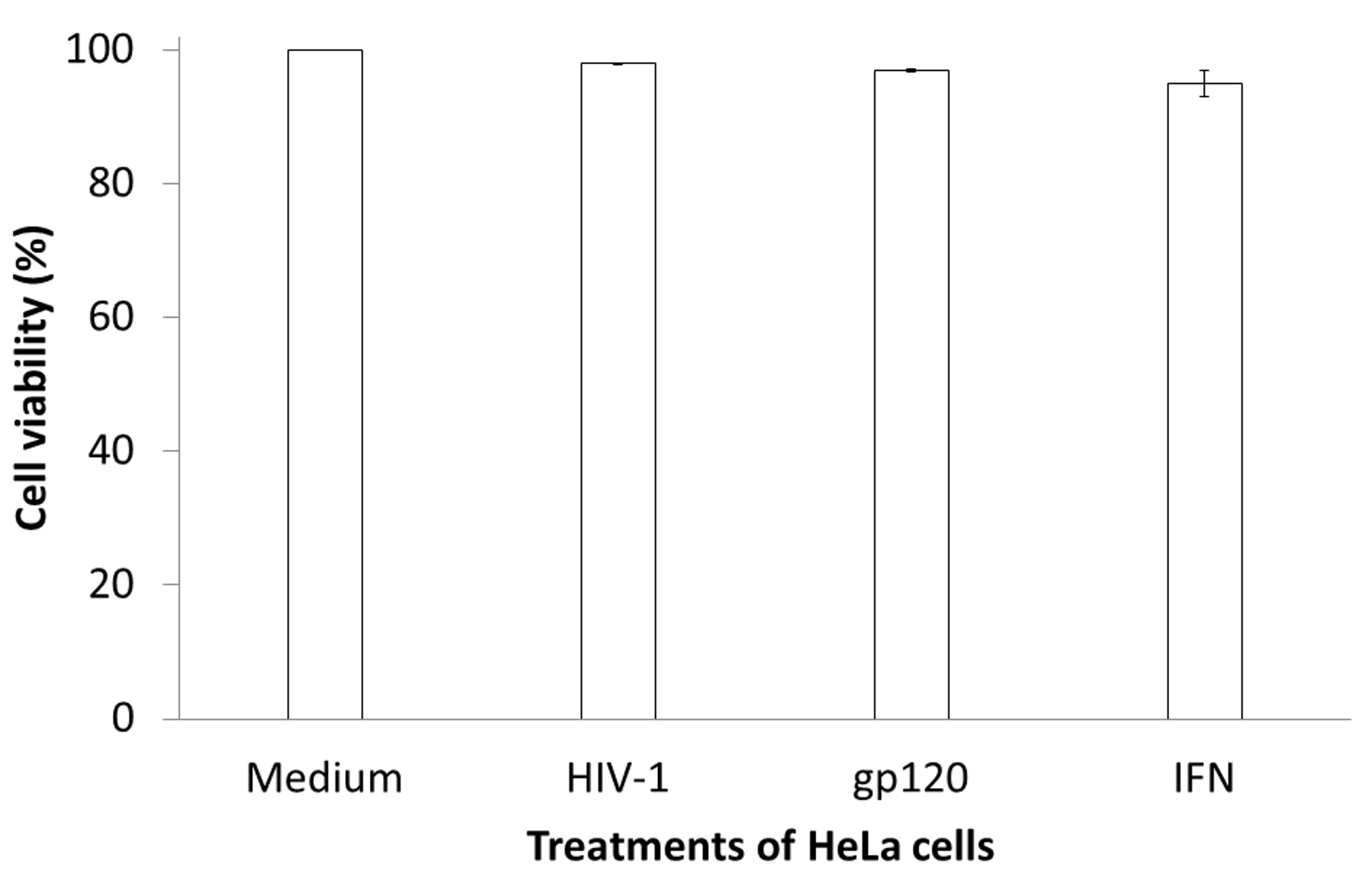

Supplement: Figure S4 — Viability of HeLa cells exposed to HIV-1. HeLa cells were exposed to HIV-1 (10 ng/mL of HIV-1 p24 Ag), gp120 (5 µg/mL) or IFN-γ (10 ng/mL). After 72 h, cell viability was measured by XTT assay. (n = 4). (TIF) [file pone.0101056.s004.tif]

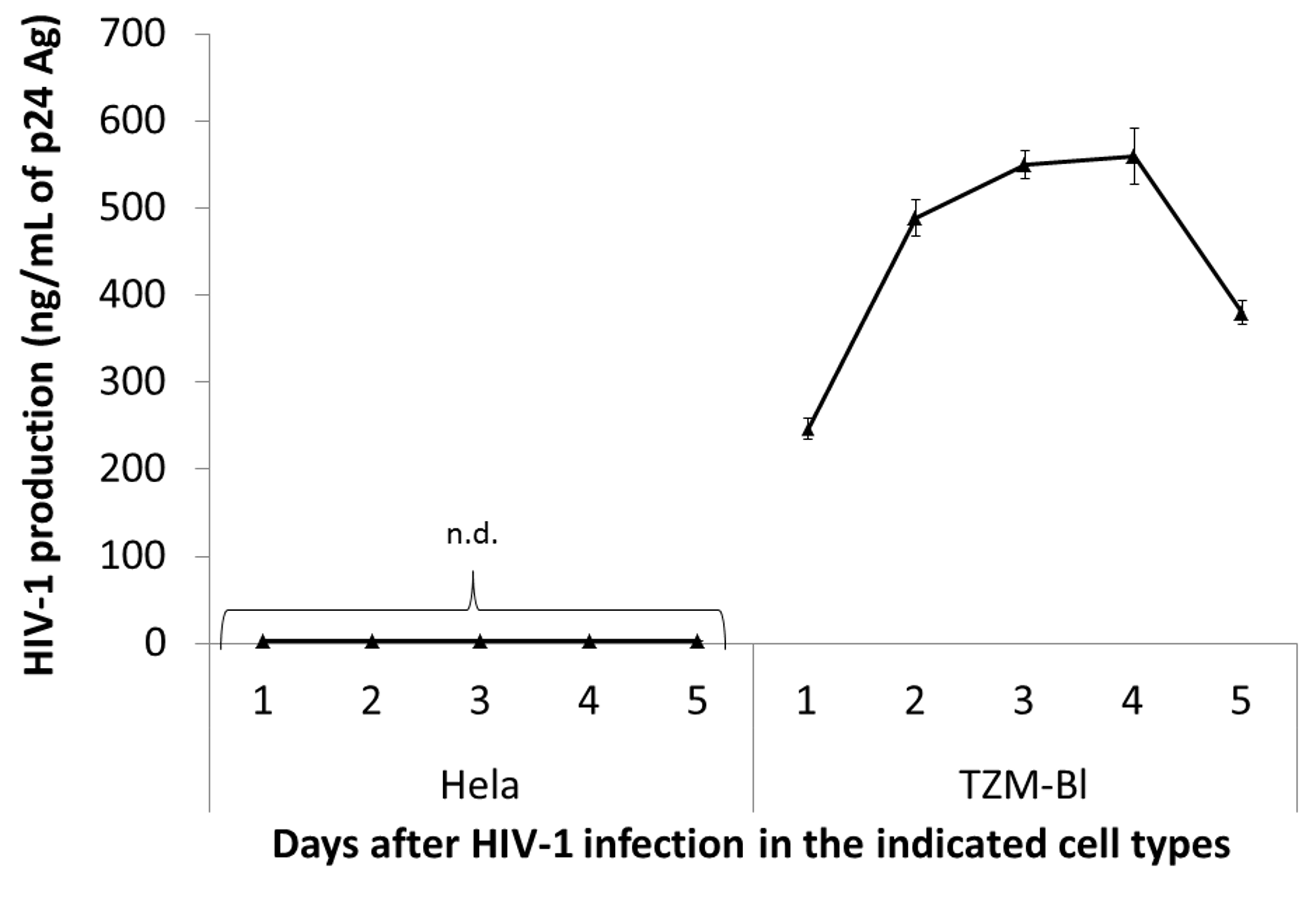

Supplement: Figure S5 — HIV-1 does not grow in HeLa cell lines. The cell lineages HeLa and TZM-bl (which has the HeLa background but expresses CD4 and CCR5, among other genes) were exposed to HIV-1 (10 ng/mL of HIV-1 p24 Ag) for 1 h at 37°C. After that, cells were washed to remove unbounded viruses and fresh culture medium was added. At indicated days after infection, aliquots of the culture supernatants were collected to measure HIV-1 p24 Ag by ELISA. HIV-1 production was not detected (n.d.) in HeLa cells. (n = 4). (TIF) [file pone.0101056.s005.tif]

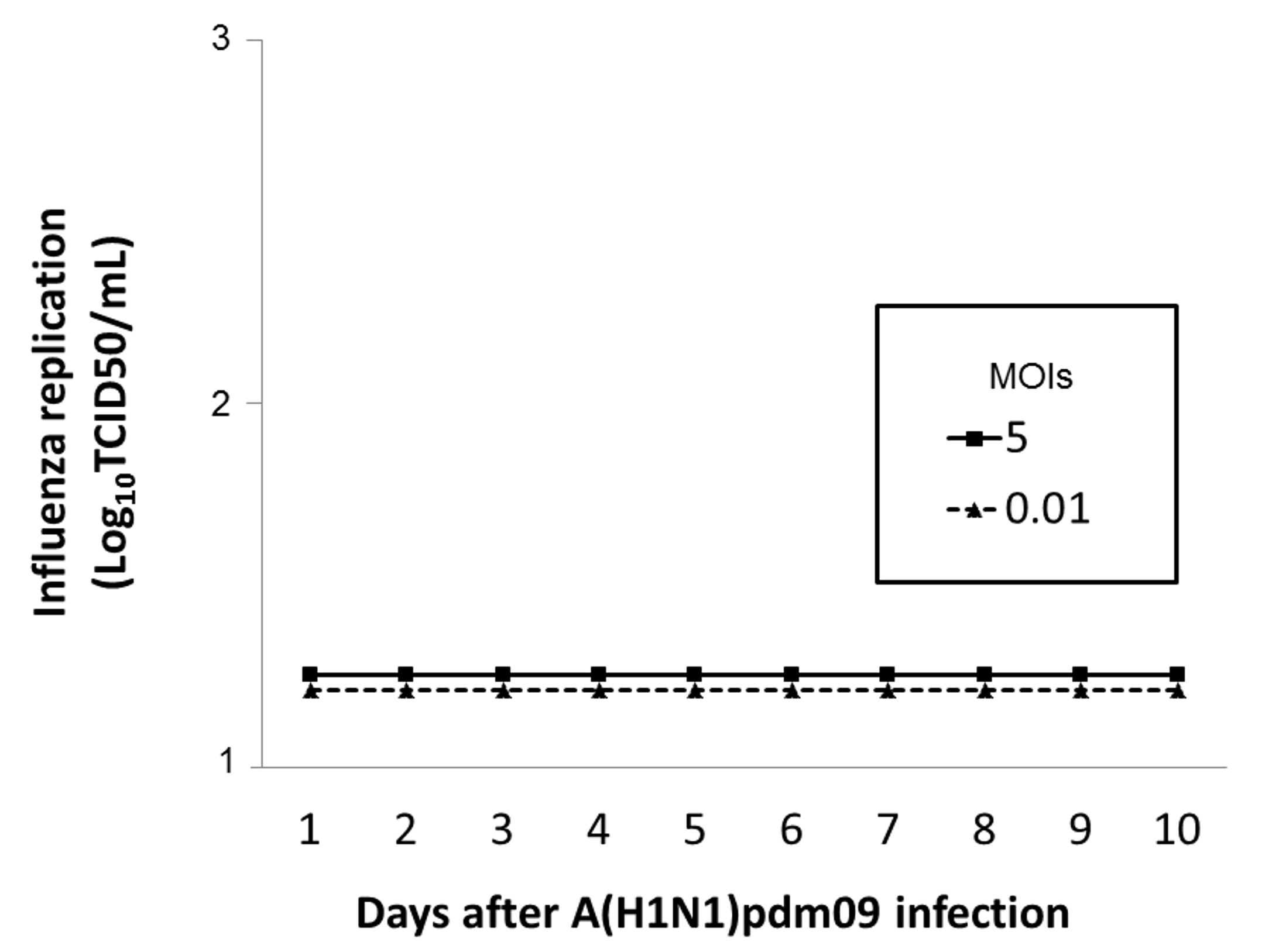

Supplement: Figure S6 — Macrophages are non-permissive to influenza A(H1N1)pdm09. Macrophages were infected with the indicated MOIs of influenza A(H1N1)pdm09 for 1 h at 37°C with inoculation medium (serum-free DMEM containing 0.2% serum albumin and trypsin at 4 µg/mL). After that, cells were washed to remove unbounded viruses and fresh inoculation medium was added. At indicated days after infection, aliquots of the culture supernatants were collected and tittered in MDCKs by end-point dilution (TCID50/mL). (n = 3). (TIF) [file pone.0101056.s006.tif]
