# Supplementary material for: HIV-1 and Its gp120 Inhibits the Influenza A(H1N1)pdm09 Life Cycle in an IFITM3-Dependent Fashion
Source: PLoS One. 2014 Jun 30;9(6):e101056. doi: 10.1371/journal.pone.0101056 (PMC4076258; doi:10.1371/journal.pone.0101056)
Supplement: Table S1 — Primer sequences. (DOCX) [file pone.0101056.s007.docx]

| **Target** | **positions** | **sequences** |
| --- | --- | --- |
| GAPDH | Forward | 5´-GCT CCT CCT GTT CGA CAG TCA-3´ |
|  | Reverse | 5´-ACC TTC CCC ATG GTG TCT GA-3´ |
| b-Actin | Forward | 5´-TGG ACA TCC GCA AAG AAC TG-3´ |
|  | Reverse | 5´- GCC GAT CCA CAC GGA GTA CTT-3´ |
| RNAseP | Forward | 5´- AGA TTT GGA CCT GCG AGC G-3´ |
|  | Reverse | 5´- GAG CGG CTG TCT CCA CAA GT-3´ |
| lncRNA | Forward | 5´- TCC TTG CTA ATA TTC TGC CCC-3´ |
|  | Reverse | 5´- TTC CTT CTT CCT CCT CAT TCT TC-3´ |

**Table S1 – Primer sequences**
