# Supplementary material for: HIV-1 and Its gp120 Inhibits the Influenza A(H1N1)pdm09 Life Cycle in an IFITM3-Dependent Fashion
Source: PLoS One. 2014 Jun 30;9(6):e101056. doi: 10.1371/journal.pone.0101056 (PMC4076258; doi:10.1371/journal.pone.0101056)
Supplement: Table S2 — Basic clinical information from HIV-1-infected individuals with laboratory-confirmed diagnosis of influenza A(H1N1)pdm09. (DOCX) [file pone.0101056.s008.docx]

| **Sample** | **State** | **Gender** | **Age** | **Beginning of symptoms** | **Sample collection** | **Epidemiological week of 2009** |
| --- | --- | --- | --- | --- | --- | --- |
|  |  |  | **(years)** |  |  |  |
| 5470 | RS | F | 23 | 18/jul/09 | 23/jul/09 | 29 |
| 5661 | RS | M | 32 | 22/jul/09 | 24/jul/09 | 29 |
| 5922 | RS | M | 33 | 26/jul/09 | 27/jul/09 | 30 |
| 6529 | RJ | F | 41 | 27/jul/09 | 30/jul/09 | 30 |
| 7313 | RS | F | 40 | 29/jul/09 | 30/jul/09 | 30 |
| 13898 | SC | F | 18 | 07/ago/09 | 14/ago/09 | 31 |
| 14529 | SC | F | 19 | 13/ago/09 | 17/ago/09 | 32 |

**Table s2 – Basic clinical information from HIV-1-infected individuals with laboratory-confirmed diagnosis of influenza A(H1N1)pdm09**
